# Making hybrid [*n*]-rotaxanes as supramolecular arrays of molecular electron spin qubits

**DOI:** 10.1038/ncomms10240

**Published:** 2016-01-08

**Authors:** Antonio Fernandez, Jesus Ferrando-Soria, Eufemio Moreno Pineda, Floriana Tuna, Iñigo J. Vitorica-Yrezabal, Christiane Knappke, Jakub Ujma, Christopher A. Muryn, Grigore A. Timco, Perdita E. Barran, Arzhang Ardavan, Richard E.P. Winpenny

**Affiliations:** 1 School of Chemistry and Photon Science Institute, The University of Manchester, Oxford Road, Manchester M13 9PL, UK; 2 Department of Chemistry, University of Oxford, Oxford OX1 3TA, UK; 3 The Michael Barber Centre for Collaborative Mass Spectrometry, Manchester Institute of Biotechnology, The University of Manchester, Oxford Road, Manchester M13 9PL, UK; 4 Department of Physics, Centre for Advanced Electron Spin Resonance, The Clarendon Laboratory, University of Oxford, Parks Road, Oxford OX1 3PU, UK

## Abstract

Quantum information processing (QIP) would require that the individual units involved—qubits—communicate to other qubits while retaining their identity. In many ways this resembles the way supramolecular chemistry brings together individual molecules into interlocked structures, where the assembly has one identity but where the individual components are still recognizable. Here a fully modular supramolecular strategy has been to link hybrid organic–inorganic [2]- and [3]-rotaxanes into still larger [4]-, [5]- and [7]-rotaxanes. The ring components are heterometallic octanuclear [Cr_7_NiF_8_(O_2_C^*t*^Bu)_16_]^–^ coordination cages and the thread components template the formation of the ring about the organic axle, and are further functionalized to act as a ligand, which leads to large supramolecular arrays of these heterometallic rings. As the rings have been proposed as qubits for QIP, the strategy provides a possible route towards scalable molecular electron spin devices for QIP. Double electron–electron resonance experiments demonstrate inter-qubit interactions suitable for mediating two-qubit quantum logic gates.

## Abstract (web summary)

Paramagnetic heterometallic rings have long been considered as possible qubits within a quantum information processing system. Here, the authors employ supramolecular chemistry to fabricate multiple rings around multi-armed threads, as an important step towards generating useful qubit arrays.

## Graphical abstract

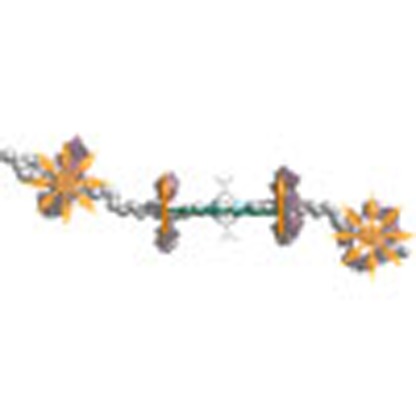

A marked trend during the last decades has been the extreme miniaturization of components in information technologies aiming to produce devices with higher speed and storage capacity. As we reach features of ∼10 nm classical physical laws cease to be dominant, and quantum devices will result as we enter the nanometre scale, with quantum mechanics governing the behaviour of the systems. Owing to the intrinsic nature of quantum systems, quantum information processing offers the possibility of performing some computational tasks far more quickly than is possible using conventional computers. These tasks include searching of unsorted directories, using the Grover algorithm^1^, and factoring large numbers in primes, using the Shor algorithm^2^. Furthermore, Lloyd proved the correctness of the conjecture made by Feynman, stating that quantum systems are most successfully simulated using other quantum systems^3^,^4^. Therefore, the motivation to produce quantum computers is considerable, and has attracted a great deal of interest from scientist working in materials science, chemistry, physics and nano-fabrication technologies. For example, the company D-Wave has reported a quantum annealer^5^ that performs certain calculations sufficiently rapid that a consortium involving Google and NASA has invested staggering sums of money in one such device. Many implementation strategies have been proposed for creating quantum computers including superconducting qubits^6^, quantum dots^7^, photons and trapped atoms^8^. Particularly relevant here are experiments involving nuclear spins, for example recent astonishing work on terbium phthalocyanine complexes^9^, where it is possible to drive quantum oscillations within single nuclei using microwaves.

A major challenge in developing devices for quantum computation is bringing together sufficient interacting units (qubits) to carry out useful algorithms. This is the major difficulty for quantum systems such as ^13^C sites near nitrogen vacancies in diamond; the individual sites have excellent performance^10^, but cannot be linked controllably into useful arrays. In an alternative approach, molecular nanomagnets have been proposed as qubits. The first molecular magnet to be proposed for quantum computing was the famous polymetallic cage {Mn_12_} (ref. 11). Since then other molecular systems have been proposed, chiefly the idea of bringing together two two-level systems to make universal quantum gates^12^,^13^,^14^,^15^,^16^,^17^,^18^,^19^,^20^. Individually, they do not have the very long phase memory times associated with defect sites, but in supramolecular chemistry^21^ a methodology exists to link together molecular qubits into entangled arrays with great control. Supramolecular chemistry brings together molecules with interactions that are weaker than covalent bonds, such that the individual units retain their individual character. In many ways, this is reminiscent of the idea for obtaining an entanglement equilibrium state with quantum mechanics; where individual components retain their identities, but an action at one unit influences another.

We have proposed using heterometallic rings as a qubit^22^, specifically looking at the {Cr_7_Ni} rings that have an 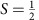 ground state as two-level systems that meet criteria required for qubits, that is, it is possible to initialize them and the energy scales are correct^22^. They also have sufficient phase memory times *T*_m_ to allow gate operation before state degradation can occur^23^, and we can control the interaction between rings to create entangled states^24^. We have also shown these rings have significant *T*_m_ values when doped into diamagnetic lattices^25^, which in the longer term brings the possibility of addressing individual transitions in orientated single crystals. Other groups have reported still longer *T*_m_ values for simpler paramagnets^26^,^27^. The chemical advantage of using the heterometallic ring is that there are many methods available, due to their vast chemical versatility, to link them together or to other chemical entities^28^,^29^,^30^,^31^. Here we propose a fully modular design strategy to create large arrays of molecular qubits and show the first steps in delivering supramolecular arrays of qubits.

## Results

### Design and synthesis of hybrid rotaxanes

**Figure 1:**
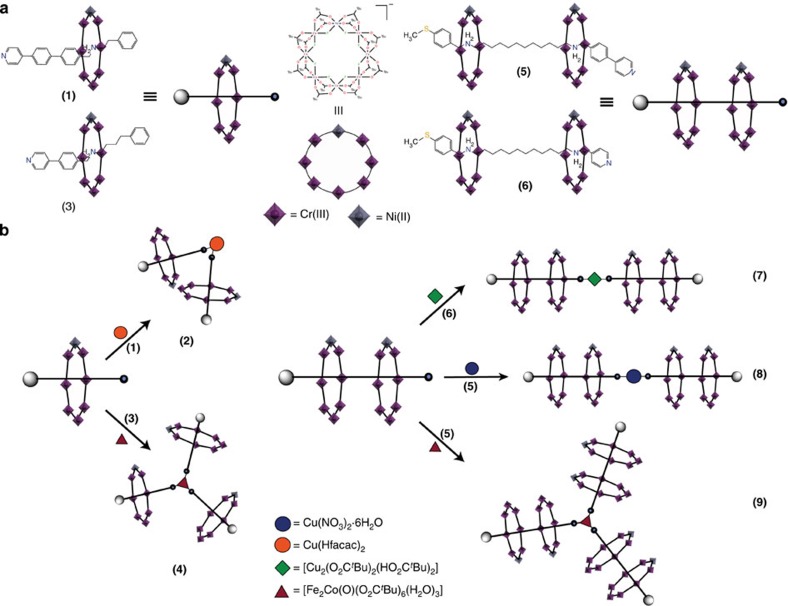
Supramolecular strategy for constructing large arrays of qubits using rotaxanes as ligands. (**a**) Scheme for the components of the larger arrays. (**b**) Reactions to give larger [*n*]-rotaxanes. The numbers in parentheses refer to compounds discussed in the text.

Conceptually what we intend involves growing a large array through combining two steps, building from our reports of hybrid organic–inorganic rotaxanes^32^,^33^. Hybrid rotaxanes contain an organic thread to bring together two or more heterometallic rings. In a first step, by means of basic concepts of organic and supramolecular chemistry, we build rotaxanes of increasing complexity, where we functionalise one end of a thread with a pyridine group. In a second step, we make use of single metal sites and coordination complexes with open coordination positions, to act as a central core and bring together very large number of qubits within a supramolecular assembly (Fig. 1).

**Figure 2:**
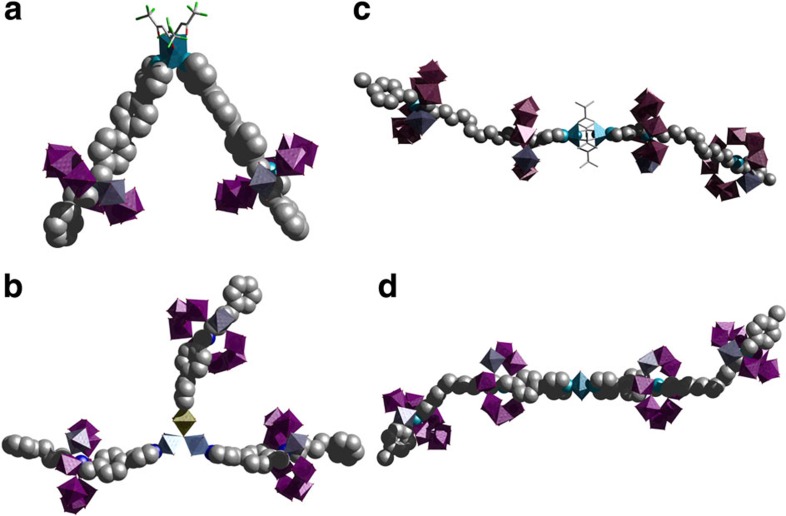
Single crystal structures of polyrotaxanes. (**a**) [3]-rotaxane (**2**); (**b**) [4]-rotaxane (**4**); (**c**) [5]-rotaxane (**7**) and (**d**) [5]-rotaxane (**8**). Hydrogen atoms and pivalate groups omitted for clarity. Metal sites shown as polyhedra. Colour code: Cu, light blue; Cr, purple; Ni, teal; N, blue; O, red; C, grey; F, green; H omitted for clarity. Crystal and refinement parameters given in Supplementary Table 1.

To show the general validity and versatility of our design strategy, firstly we synthesized the [2]-rotaxane [**A**H_2_{Cr_7_Ni(μ-F)_8_(O_2_C^*t*^Bu)_16_}_2_] (**1**), that contains a pyridine group at the edge of the thread, from the reaction of 2-phenyl-*N*-{[4′-(pyridin-4-yl)-(1,1′-biphenyl)-4-yl]methyl}ethanamine **A** (ref. 34), chromium fluoride (CrF_3_·4H_2_O) and a nickel pivalate salt [Ni_2_(μ-OH_2_)(O_2_C^*t*^Bu)_4_(HO_2_C^*t*^Bu)_4_] (ref. 35) (1:6:2 molar ratio) using pivalic acid as solvent with a moderate yield (10%). Subsequently reaction of the [2]-rotaxane **1** with hydrated [Cu(hfacac)_2_]·*n*H_2_O (2:1 molar ratio) yields the [3]-rotaxane [*cis*-{Cu(hfacac)_2_}**A**H_2_{Cr_7_Ni(μ-F)_8_(O_2_C^*t*^Bu)_16_}_2_] (**2**) (Fig. 2a), with three potential qubits (the single Cu^II^ ion is also a two-level system), which contains two heterometallic rings above the central copper, with a *cis* geometry (see Supplementary Methods for synthetic detail).

To increase the number of qubits in an assembly further we can bind the [2]-rotaxane to a coordination complex that possesses more than two open coordination positions. To this end, we prepared thread **B**, 4-phenyl-*N*-(4-(pyridin-4-yl)benzyl)butan-1-amine^36^, and make the [2]-rotaxane [**B**H_2_{Cr_7_Ni(μ-F)_8_(O_2_C^*t*^Bu)_16_}] (**3**). Reaction of **3** with [Fe_2_Co(μ_3_-O)(O_2_C^*t*^Bu)_6_(H_2_O)_3_] (ref. 37) (3:1 molar ratio) gives a triangular [4]-rotaxane [Fe_2_Co(μ_3_-O)(O_2_C^*t*^Bu)_6_{**B**H_2_{Cr_7_Ni(μ-F)_8_(O_2_C^*t*^Bu)_16_}}_3_] (**4**), with three qubits, where the terminal water molecules of the metal carboxylate triangle have been replaced by pyridine–nitrogen atoms of three [2]-rotaxane (Fig. 2b).

Another route to increase the number of qubits in the supramolecular array is to increase the number of heterometallic rings in each individual rotaxane. With this in mind, we synthesized threads **C**, *N*^1^-(4-(methylthio)benzyl)-*N*^12^-(4-(pyridin-4-yl)benzyl)dodecane-1,12-diamine and **D**, *N*^1^-(4-(methylthio)benzyl)-*N*^12^-(pyridin-4-ylmethyl)dodecane-1,12-diamine^38^, in good yield (Supplementary Methods). Reaction of **C** with CrF_3_·4H_2_O and [Ni_2_(μ-OH_2_)(O_2_C^*t*^Bu)_4_(HO_2_C^*t*^Bu)_4_] (1:14:4.5 molar ratio) with pivalic acid as solvent, leads to a [3]-rotaxane with formula [**C**H_2_{Cr_7_Ni(μ-F)_8_(O_2_C^*t*^Bu)_16_}_2_] (**5**) in 23% yield, while reaction with **D** gives [3]-rotaxane [**D**H_2_{Cr_7_Ni(μ-F)_8_(O_2_C^*t*^Bu)_16_}_2_] (**6**) in 60% yield (**6**). These yields are good given the complex assembly process involved. The reactions in each case protonate both secondary amines and a {Cr_7_Ni} ring grows around each, leaving the pyridine group available for further coordination. Addition of **6** or **5** with [Cu_2_(O_2_C^*t*^Bu)_4_(HO_2_C^*t*^Bu)_2_] and Cu(NO_3_)_2_·6H_2_O, respectively (2:1 molar ratio) in hot toluene, allow us to obtain [{Cu(O_2_C^*t*^Bu)_2_}**D**H_2_{Cr_7_Ni(μ-F)_8_(O_2_C^*t*^Bu)_16_}_2_]_2_ (**7**) and [{Cu(NO_3_)_2_}{**C**H_2_{Cr_7_Ni(μ-F)_8_(O_2_C^*t*^Bu)_16_}_2_}_2_] (**8**) (Fig. 2c,d). Compounds **7** and **8** are [5]-rotaxanes that are almost 7 nm long; they are formed in an overall yield of 70 (**7**) and 75% (**8**) based on thread **C** and **D**, respectively. These [5]-rotaxanes contain four {Cr_7_Ni} rings; in **8** the presence of a paramagnetic Cu^II^ ion within the adds a further qubit, producing a [5]-rotaxane containing five qubits.

The final step of the strategy is to combine these two routes to increase the number of heterometallic rings per assembly. In this sense, **5** was reacted with the central core [Fe_2_Co(μ_3_-O)(O_2_C^*t*^Bu)_6_(H_2_O)_3_] (3:1 molar ratio) in hot acetone, with the aim to get a [7]-rotaxane of formula [{Fe_2_Co(μ_3_-O)(O_2_C^*t*^Bu)_6_}{**C**H_2_{Cr_7_Ni(μ-F)(O_2_C^*t*^Bu)_16_}_2_}_3_] (**9**). Unfortunately, while compounds **2**, **4**, **7** and **8** yield X-ray quality crystals, we were unable to get suitable crystals of **9** for single-crystal X-ray diffraction experiment.

### Characterization of a [7]-rotaxane

**Figure 3:**
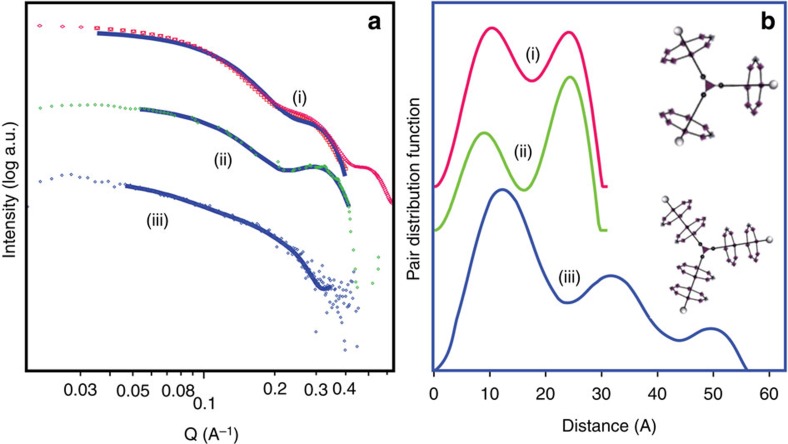
SAXS on [4]- and [7]-rotaxanes. (**a**) SAXS data; (i) calculated diffraction from crystallographically determined structure of **4**, (ii) experimental data from **4**, (iii) experimental data from **9**. The solid line in each spectra is the fit that is associated with the pair distribution function plots shown in **b**. (**b**) Pair distance distribution function; (i) fit to calculated SAXS data for **4**, (ii) fit to experimental data collected for **4** and (iii) fit to experimental data collected for **9**. Inset: schematic representation of **4** (top) and **9** (bottom). The pair distance distribution function curves presented were also correlated with Guinier plots and gyration radius calculations to ensure consistency within the data analysis.

In the absence of a crystal structure, and because of the large number of paramagnetic centres present in **9** (that makes NMR spectroscopy less useful than for larger organic molecules), we used small angle X-ray scattering (SAXS) to demonstrate that the [7]-rotaxane has been made. SAXS has been used to characterize porphyrin arrays^39^,^40^,^41^, which present the same problems as **9**. Owing to poor sample solubility, saturated solutions were employed in the SAXS collections. To ensure no aggregation had occurred, diluted collections were also performed and compared (see Supplementary Figures 11–17). We began by examining compound **4**, where we have a full crystal structure determination and which can be considered a small version of **9**. Here SAXS data shows two broad maxima at 12 and 22 Å, which match perfectly with the distance obtained by X-ray crystallography in an individual [2]-rotaxane from the central core to the centre of the heterometallic ring and the size of the assembly, respectively (Fig. 3).

**Figure 4:**
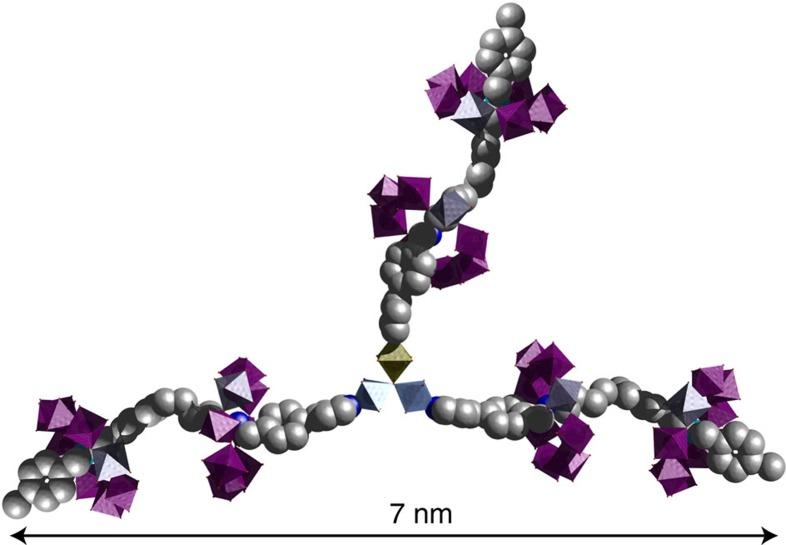
Proposed structure for [7]-rotaxane **9**. The proposed formula for this compound is [Fe_2_Co(μ_3_-O)(O_2_C^*t*^Bu)_6_{**C**H_2_{Cr_7_Ni(μ-F)(O_2_C^*t*^Bu)_16_}_2_}_3_]; colour code as Fig. 2.

Then we move to compound **9**, where we have no structure. The first remarkable feature that we noticed was a significant change in the X-ray scattering compare with **4**, as we expected owing to the larger number of heterometallic rings in this supramolecular array. Interestingly, the analysis of the diffraction data with pair distance distribution function shows pairs of particles to much larger distances, with the maximum extent being ca. 55 Å and three maxima at 12, 32 and 50 Å. It can be speculated that the 12 Å distance is related to contacts within a single ring and is consistent with the data from the [4]-rotaxane. The 32 and 50 Å could then be related to distances between one inner ring with an outer ring on a different arm and an outer ring to outer ring distance. The model is therefore consistent with six {Cr_7_Ni} rings about a central {Fe_2_Co} triangle, and matches the proposed structure for **9** (Fig. 4).

We attempted to derive further evidence for the formation of **9** from electrospray mass spectrometry, however this [7]-rotaxane did not fly as an intact species under mass spectrometry conditions. However, we prepared an analogous version of **9** with Zn(II) [{Fe_2_Co(μ_3_-O)(O_2_C^*t*^Bu)_6_}{**C**H_2_{Cr_7_Zn(μ-F)(O_2_C^*t*^Bu)_16_}_2_}_3_] (**10**) and this shows a peak for **10** for a trication at *m*/*z*=5,188; matching well with the calculated mass of 15,424 (see Supplementary Methods and Supplementary Figures 6–10 for further details).

### Hybrid rotaxanes in quantum logic gates

A key question remains whether it is possible to produce and control interactions between qubits that would allow operation of quantum logic gates. The heterometallic ring monomers discussed here can exhibit phase memory times (*T*_m_) exceeding 10 μs (ref. 23), although more typically they are a little below 1 μs (refs 25, 42). A single-qubit manipulation using commercial electron paramagnetic resonance (EPR) apparatus takes of the order of 10 ns. Any two-qubit interaction capable of, for example, generating controlled entanglement, should do so on a timescale intermediate between the single-qubit manipulation time and the phase memory time, that is, a gate time of 100–300 ns.

A major concern has been that in complex molecules featuring multiple weakly interacting spins, this will introduce a source of decoherence, and therefore significantly decrease the value of *T*_m_. However, a recent study of a molecule containing twenty-four qubits suggests this is not a significant problem^31^. To confirm this, we have studied *T*_m_ for compounds **2**, **4**, **5** and **7** using pulsed EPR spectroscopy (see Supplementary Methods for experimental details); in **2**, **5** and **7** we can fit the measurements to a stretched exponential with *T*_m_=795 ns and a stretch parameter of 1.7. These values are similar to many other heterometallic rings containing pivalate ligands^23^,^25^. In compound **4** *T*_m_=700 ns with a similar stretch parameter. These results confirm that bringing together multi-qubit assemblies will not have a deleterious effect on the phase memory.

To examine the interaction between the qubits we have used double electron–electron resonance (DEER) spectroscopy^43^, which is established in structural biology as a way of measuring distances through the strength of the dipolar interaction between spins. Here we are interested in the two-qubit gate time, which comes directly out of the DEER experiment (see SI for experimental details, Supplementary Figures 1–5 and Supplementary Table 2). The experiment involves flipping the spin on one centre and examining the resulting precession of a second spin under the change in its effective local magnetic field. The time taken for half of a complete precession corresponds directly to the duration of a two-qubit conditional phase gate (one of the fundamental entangling gates)^44^.

**Figure 5:**
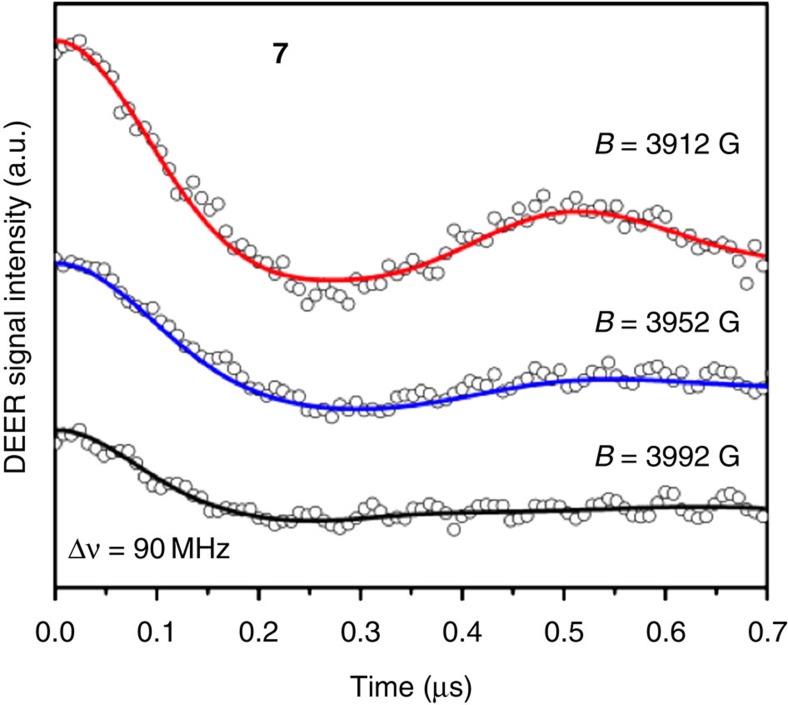
DEER data for compound **7**. Samples were measured in toluene at 2.6 K for three values of the magnetic field within the resonance line (3,912, 3,952 and 3,992 G). The probe and pump frequencies were *υ*_1_=9.7648 GHz and *υ*_2_=*υ*_1_–90 MHz, respectively. See Supplementary Methods for more experimental details.

Figure 5 shows typical DEER data from compound **7**, for several different magnetic fields within the EPR absorption spectrum. Compound **7** was studied because the central ring…ring interaction involves a rigid thread with the two rings co-parallel. This makes interpretation of the DEER data more straightforward than in cases where the thread is flexible or where the rings are not co-parallel. A large and oscillatory modulation of the DEER signal as a function of the time position of the pump pulse is visible for a field of 3,912 G; this oscillation represents the precession of the probe spin in the effective magnetic field of the pump spin, yielding a two-qubit gate time of ∼260 ns consistent with a dipolar interaction between the qubits. Weaker modulations occur for the higher magnetic fields of 3,952 G and 3,992 G. The field dependence arises because different orientational subpopulations are excited at each magnetic field, owing to the anisotropy of the monomers' g factors^44^.

This two-qubit gate time falls between the operation time for a single qubit and the phase memory time *T*_m_, matching observations we have made in simpler [3]-rotaxanes^44^. This establishes the presence of two-qubit interactions that are of the right strength for implementing quantum logic gates within assemblies of heterometallic rings. Studies of the other rotaxanes reported here are progressing.

## Discussion

In summary, we have shown the general validity of our modular design strategy to overcome one of the major challenges that face quantum computation to bring together many potential qubits. Using simple concepts from coordination and supramolecular chemistry we have shown a gradual increase of the complexity in the arrays synthesized. These results are the first steps of this new strategy to increase the number of qubits in a supramolecular array.

The longer perspective is how such supramolecular compounds could be used as multi-qubit arrays. First, an array of qubits could be prepared in an initial state by cooling the sample in the presence of a moderate magnetic field. This is as the relevant energy scale for preparation of an initial state is the Zeeman energy of the individual electron spins. The inter-qubit interaction energy scale is much smaller, therefore if we can prepare one qubit by cooling in field, we can prepare a multi-qubit array in the same way. This differs from nuclear spins, where the Zeeman energy is much smaller. The major remaining challenge is then to achieve local control to address individual pairs of electron spins to perform selected qubit gates. The DEER results reported above and elsewhere^44^ suggest this should be possible. The EPR spectra of these compounds are strongly anisotropic and hence it is only at specific orientations where the spin on one qubit interacts with the spin on a second. Studies of orientated single crystals of multi-qubit arrays are planned for the future where we will attempt to demonstrate how such selectivity can be used to perform simple two-qubit gates.

## Methods

### Synthesis

Unless stated otherwise, all reagents and solvents were used without further purification. The syntheses of the hybrid organic–inorganic rotaxanes were carried out in Erlenmeyer Teflon FEP flasks supplied by Fisher. Column chromatography was carried out using Silica 60A (particle size 35–70 μm, Fisher, UK) as the stationary phase, and TLC was performed on precoated silica gel plates (0.25 mm thick, 60 F254, Merck, Germany) and observed under ultraviolet light. NMR spectra were recorded on Bruker AV 400 and Bruker DMX 500 instruments. Chemical shifts are reported in p.p.m. from low to high frequency and referenced to the residual solvent resonance. electrospray ionization mass spectrometry, matrix-assisted laser desorption ionization-time of flight spectrometry and microanalysis were carried out by the services at the University of Manchester. Details of the synthetic procedures are given in the Supplementary Methods.

Details of the physical characterization and DEER studies are also given in the Supplementary Methods.

## Additional information

**Accession codes**: The X-ray crystallographic coordinates for structures reported in this study have been deposited at the Cambridge Crystallographic Data Centre, under deposition numbers 1037544–1037547. These data can be obtained free of charge from The Cambridge Crystallographic Data Centre via www.ccdc.cam.ac.uk/data_request/cif.

**How to cite this article:** Fernandez, A. *et al*. Making hybrid [*n*]-rotaxanes as supramolecular arrays of molecular electron spin qubits. *Nat. Commun.* 7:10240 doi: 10.1038/ncomms10240 (2016).

## Supplementary Material

Supplementary Information Supplementary Figures 1-17, Supplementary Tables 1-2, Supplementary Methods and Supplementary References

Supplementary Data 1 Crystallographic information file for structure 2

Supplementary Data 2 Crystallographic information file for structure 4

Supplementary Data 3 Crystallographic information file for structure 7

Supplementary Data 4 Crystallographic information file for structure 8

## References

[b1] Grover L. K. Quantum computers can search arbitrarily large databases by a single query. Phys. Rev. Lett. 79, 4709–4712 (1997).

[b2] Shor P. W. Polynomial-time algorithms for prime factorization and discrete logarithms on a quantum computer. SIAM J. Comput. 26, 1484–1509 (1997).

[b3] Lloyd S. Universal quantum simulators. Science 273, 1073–1078 (1996). 8688088 10.1126/science.273.5278.1073

[b4] Feynman R. P. Simulating physics with computers. Int. J. Theor. Phys. 21, 467–488 (1982).

[b5] Johnson M. W. . Quantum annealing with manufactured spins. Nature 473, 194–198 (2011). 21562559 10.1038/nature10012

[b6] DiCarlo L. . Preparation and measurement of three-qubit entanglement in a superconducting qubit. Nature 467, 547–578 (2010). 10.1038/nature09416 20882013

[b7] Elzerman J. M. . Single-shot read-out of an individual electron spin in a quantum dot. Nature 430, 431–435 (2004). 15269762 10.1038/nature02693

[b8] Ladd T. D. . Quantum computers. Nature 464, 45–53 (2010). 20203602 10.1038/nature08812

[b9] Thiele S. . Electrically driven nuclear spin resonance in single-molecule magnets. Science 344, 1135–1138 (2014). 24904159 10.1126/science.1249802

[b10] Maurer P. C. . Room-temperature quantum bit memory exceeding one second. Science 336, 1283–1286 (2012). 22679092 10.1126/science.1220513

[b11] Leuenberger M. & Loss D. Quantum computing in molecular magnets. Nature 410, 789–793 (2001). 11298441 10.1038/35071024

[b12] Meier F., Levy J. & Loss D. Quantum computing with spin cluster qubits. Phys. Rev. Lett. 90, 47901–47904 (2003). 10.1103/PhysRevLett.90.047901 12570460

[b13] Lehmann J., Gaita-Ariño A., Coronado E. & Loss D. Spin qubits with electrically gated polyoxometalate molecules. Nat. Nanotechnol. 2, 312–317 (2007). 18654290 10.1038/nnano.2007.110

[b14] Sañudo E. C. . Molecules composed of two weakly magnetically coupled [Mn_4_^III^] clusters. Inorg. Chem. 46, 9045–9047 (2007). 17900188 10.1021/ic701467y

[b15] Santini P., Carretta S., Troiani F. & Amoretti G. Molecular nanomagnets as quantum simulators. Phys. Rev. Lett. 107, 230502 (2011). 22182075 10.1103/PhysRevLett.107.230502

[b16] Troiani F. & Affronte M. Molecular spins for quantum information technologies. Chem. Soc. Rev. 40, 3119–3129 (2011). 21336365 10.1039/c0cs00158a

[b17] Nakazawa S. . Synthetic two-spin quantum bit: *g*-engineered exchange-coupled biradical designed for controlled-NOT gate operations. Angew. Chem. Int. Ed. 51, 9860–9864 (2012). 10.1002/anie.201204489 22936609

[b18] Aromí G., Aguilà D., Gamez P., Luis F. & Roubeau O. Design of magnetic coordination complexes for quantum computing. Chem. Soc. Rev. 41, 537–546 (2012). 21818467 10.1039/c1cs15115k

[b19] Plant S. R. . A two-step approach to the synthesis of N@C_60_ fullerene dimers for molecular qubits. Chem. Sci. 4, 2971–2975 (2013).

[b20] Aguilà D. . Heterodimetallic [LnLn'] lanthanide complexes: toward a chemical design of two-qubit molecular spin quantum gates. J. Am. Chem. Soc. 136, 14215–14222 (2014). 25203521 10.1021/ja507809w PMC4195387

[b21] Lehn J.-M. Supramolecular Chemistry: Concepts and Perspectives Wiley-VCH (1995).

[b22] Troiani F. . Molecular engineering of antiferromagnetic rings for quantum computation. Phys. Rev. Lett. 94, 207208 (2005). 16090284 10.1103/PhysRevLett.94.207208

[b23] Wedge C. J. . Chemical engineering of molecular qubits. Phys. Rev. Lett. 108, 107204 (2012). 22463450 10.1103/PhysRevLett.108.107204

[b24] Candini A. . Entanglement in supramolecular spin systems. Phys. Rev. Lett. 104, 037203 (2010). 20366678 10.1103/PhysRevLett.104.037203

[b25] Moro F. . Coherent electron spin manipulation in a dilute oriented ensemble of molecular nanomagnets: pulsed EPR on doped single crystals. Chem. Commun. 50, 91–93 (2014). 10.1039/c3cc46326e 24187686

[b26] Warner W. . Potential for spin-based information processing in a thin-film molecular semiconductor. Nature 503, 504–508 (2013). 24162849 10.1038/nature12597

[b27] Bader K. . Room temperature quantum coherence in a potential molecular qubit. Nat. Commun. 5, 5304 (2014). 25328006 10.1038/ncomms6304

[b28] Timco G. A. . Engineering the coupling between molecular spin qubits by coordination chemistry. Nat. Nanotechnol. 4, 173–178 (2008). 19265847 10.1038/nnano.2008.404

[b29] Whitehead G. F. S. . A ring of rings and other multicomponent assemblies of cages. Angew. Chem. Int. Ed. 52, 9932–9935 (2013). 10.1002/anie.201304817 23904348

[b30] Whitehead G. F. S. . The acid test: the chemistry of carboxylic acid functionalised {Cr_7_Ni} rings. Chem. Sci. 5, 235–239 (2014).

[b31] Ferrando-Soria J. . Controlled synthesis of nanoscopic metal cages. J. Am. Chem. Soc. 137, 7644–7647 (2015). 26046388 10.1021/jacs.5b04664

[b32] Lee C.-F. . Hybrid organic-inorganic rotaxanes and molecular shuttles. Nature 458, 314–318 (2009). 19295605 10.1038/nature07847

[b33] Ballesteros B. . Synthesis, structure and dynamic properties of hybrid organic-inorganic rotaxanes. J. Am. Chem. Soc. 132, 15435–15444 (2010). 20929228 10.1021/ja1074773

[b34] Cunningham P. D. & Hayden L. M. Carrier dynamics resulting from above and below gap excitation of P3HT and P3HT/PCBM investigated by optical-pump terahertz-probe spectroscopy. J. Phys. Chem. C 112, 7928–7935 (2008). 10.1021/jp906454g 19886608

[b35] Chaboussant G. . Nickel pivalate complexes: structural variations and magnetic susceptibility and ineslatic neutron scattering studies. Dalton Trans. 2758–2766 (2004). 15514763 10.1039/b406112h

[b36] You Y. . Micromolding of a highly fluorescent reticular coordination polymer: solvent-mediated reconfigurable polymerization in a soft lithographic mold. Angew. Chem. Int. Ed. 49, 3757–3761 (2010). 10.1002/anie.201000096 20401884

[b37] Abdulwahab K. O. . A one pot synthesis of monodispersed cobalt and manganese ferrite nanoparticles from bimetallic pivalate clusters. Chem. Mater. 26, 999–1013 (2014).

[b38] Rath H. . Studies of hybrid organic–inorganic [2]- and [3]-rotaxanes bound to Au surfaces. Chem. Commun. 49, 3404–3406 (2013). 10.1039/c3cc38699f 23508202

[b39] Sprafke J. K. . Belt-shaped π-systems: relating geometry to electronic structure in a six-porphyrin nanoring. J. Am. Chem. Soc. 133, 17262–17273 (2011). 21939246 10.1021/ja2045919

[b40] Kelley R. F. . Intramolecular energy transfer within butadiyne-linked chlorophyll and porphyrin dimer-faced, self-assembled prisms. J. Am. Chem. Soc. 130, 4277–4284 (2008). 18327918 10.1021/ja075494f

[b41] Tiede D. M., Zhang R., Chen L. X., Yu L. & Lindsey J. S. Structural characterization of modular supramolecular architectures in solution. J. Am. Chem. Soc. 126, 14054–14062 (2004). 15506769 10.1021/ja048209q

[b42] Kaminski D. . Quantum spin coherence in halogen-modified Cr_7_Ni molecular nanomagnets. Phys. Rev. B 90, 184419 (2014).

[b43] Schiemann O. & Prisner T. F. Long-range distance determinations in biomacromolecules by EPR spectroscopy. Q. Rev. Biophys. 40, 1–53 (2007). 17565764 10.1017/S003358350700460X

[b44] Ardavan A. . Precise control of two-qubit gate times for supramolecular molecular spin qubits. npj Quantum Inf 1, 15012 (2015).

