# Supplementary material for: Making hybrid [n]-rotaxanes as supramolecular arrays of molecular electron spin qubits
Source: Nat Commun. 2016 Jan 8;7:10240. doi: 10.1038/ncomms10240 (PMC4729860; doi:10.1038/ncomms10240)
Supplement: Supplementary Information — Supplementary Figures 1-17, Supplementary Tables 1-2, Supplementary Methods and Supplementary References [file ncomms10240-s1.pdf]

Supplementary Figures

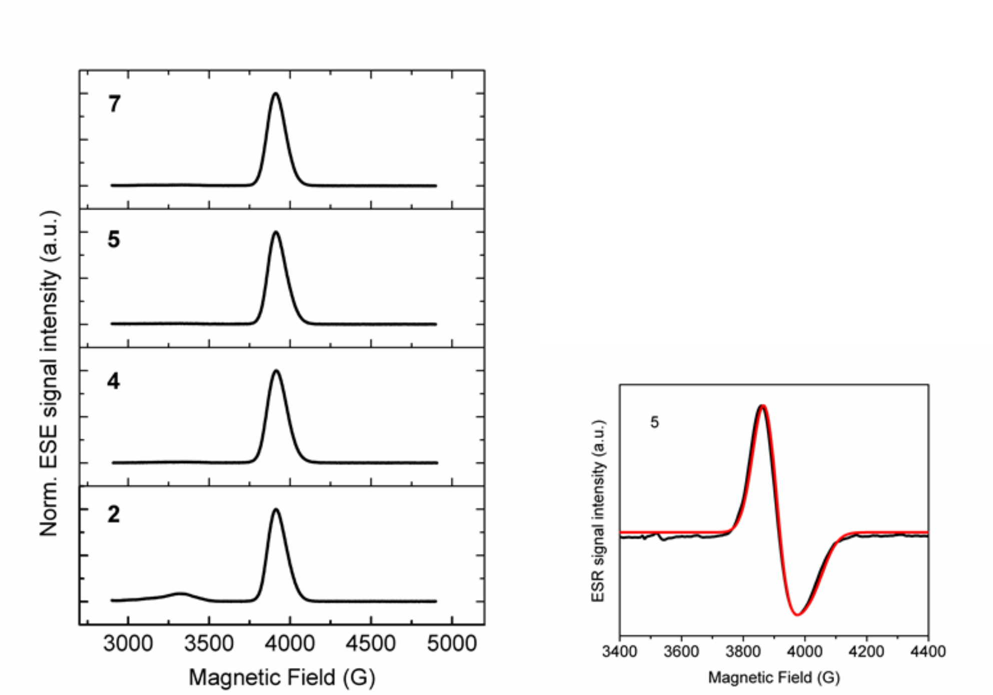

Supplementary Figure 1. Left: Echo-detected field-swept spectra at X-band and 2.6 K for **2, 4, 5** and **7** in toluene, recorded with a primary echo sequence π/2-τ-π-τ-*echo* with π = 32 ns and τ = 300 ns. **Right**: X-band EPR spectrum at 5 K for **5** (black), and simulation with $g_{\perp}$ = 1.778 and g_{II} = 1.720 (red).

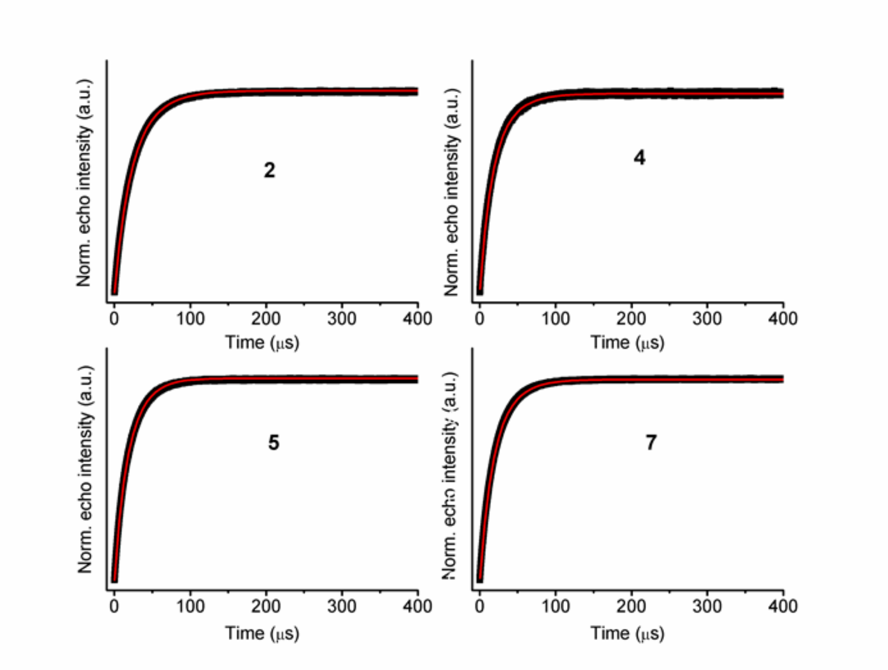

Supplementary Figure 2. Electron-spin-echo (ESE) inversion recovery curves for **2**, **4**, **5** and **7** recorded at 2.6 K and B = 3912 G (maximum resonance). The red line is a fit to the exponential decay function $I(\mathrm{t}) = I_1 \exp(-t/T_1) + I_{\mathrm{SD}} \exp(-t/T_{\mathrm{SD}})$ from which the time constants T_1 and T_{SD} were deducted (see Table S1).

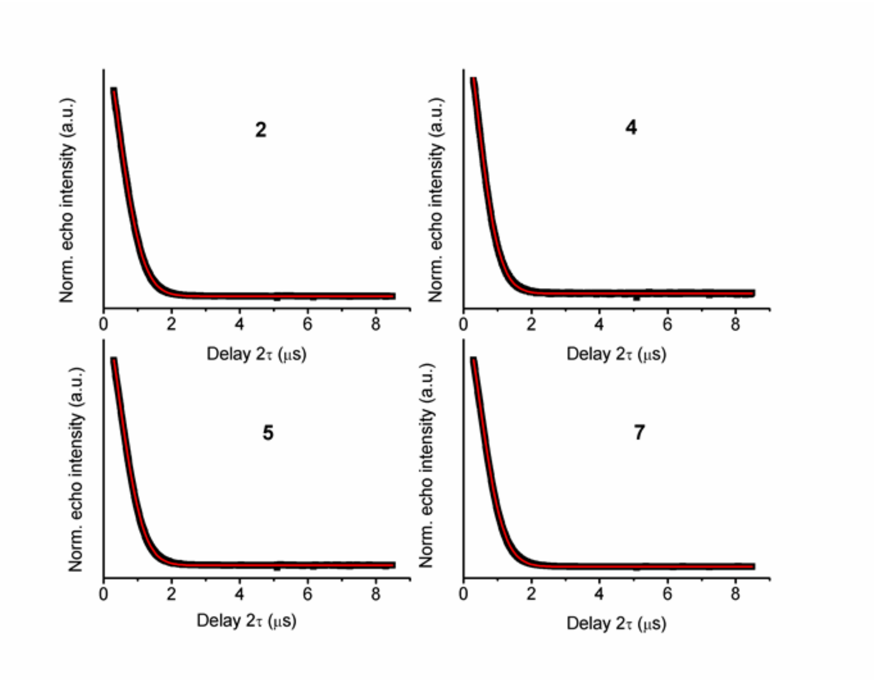

Supplementary Figure 3. Two pulse electron spin-echo decays for **2**, **4**, **5** and **7** in toluene at 2.6 K and B = 3912 G , recorded with pulses of π = 128 ns. The red lines represent fits to eq. (2), $I(2\tau)=I(0)\exp[(-2\tau/T_M)^s]$, with T_M and s values listed in Table S1.

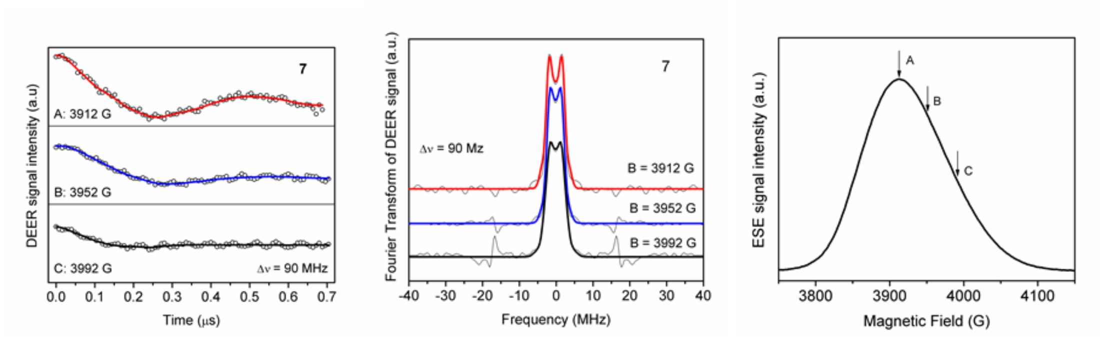

Supplementary Figure 4. DEER data for compound **7** in toluene at 2.6 K. The detection and excitation frequencies were ν_1 = 9.7648GHz and ν_2 = ν1-90MHz, respectively. The DEER signal was recorded as a function of the time of the ν_1 pump pulse (left panel) at three magnetic fields, each corresponding to an orientational sub-population based on the anisotropic nature of the ESR signal. The Fourier transforms of the time- domain data are shown in the middle panel. The right panel shows the echo-detected field-sweep spectrum of the ring-qubit with A, B, and C indicating the spectral positions for DEER excitation. At position A, rings with effective g-factors close to $g_\perp$ are excited; as the magnetic field increases, the ring orientations selected approach those with effective g-factors near $g_\parallel$.

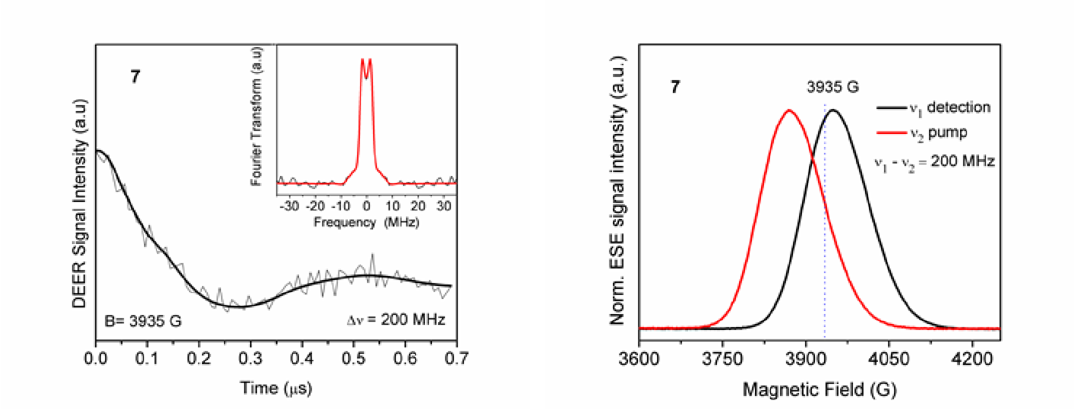

Supplementary Figure 5. **Left**: DEER data for compound **7** in toluene at 2.6 K, recorded at a magnetic field of 3935 G at detection and pump frequencies of ν_1 = 9.7648GHz and ν_2 = ν_1-200MHz respectively. The inset represents the Fourier transform of the DEER signal (experimental: black; calculation: red). **Right:** The echo-detected field sweep spectra of **7** recorded at ν_1 (black) and ν_2 (red) frequencies.

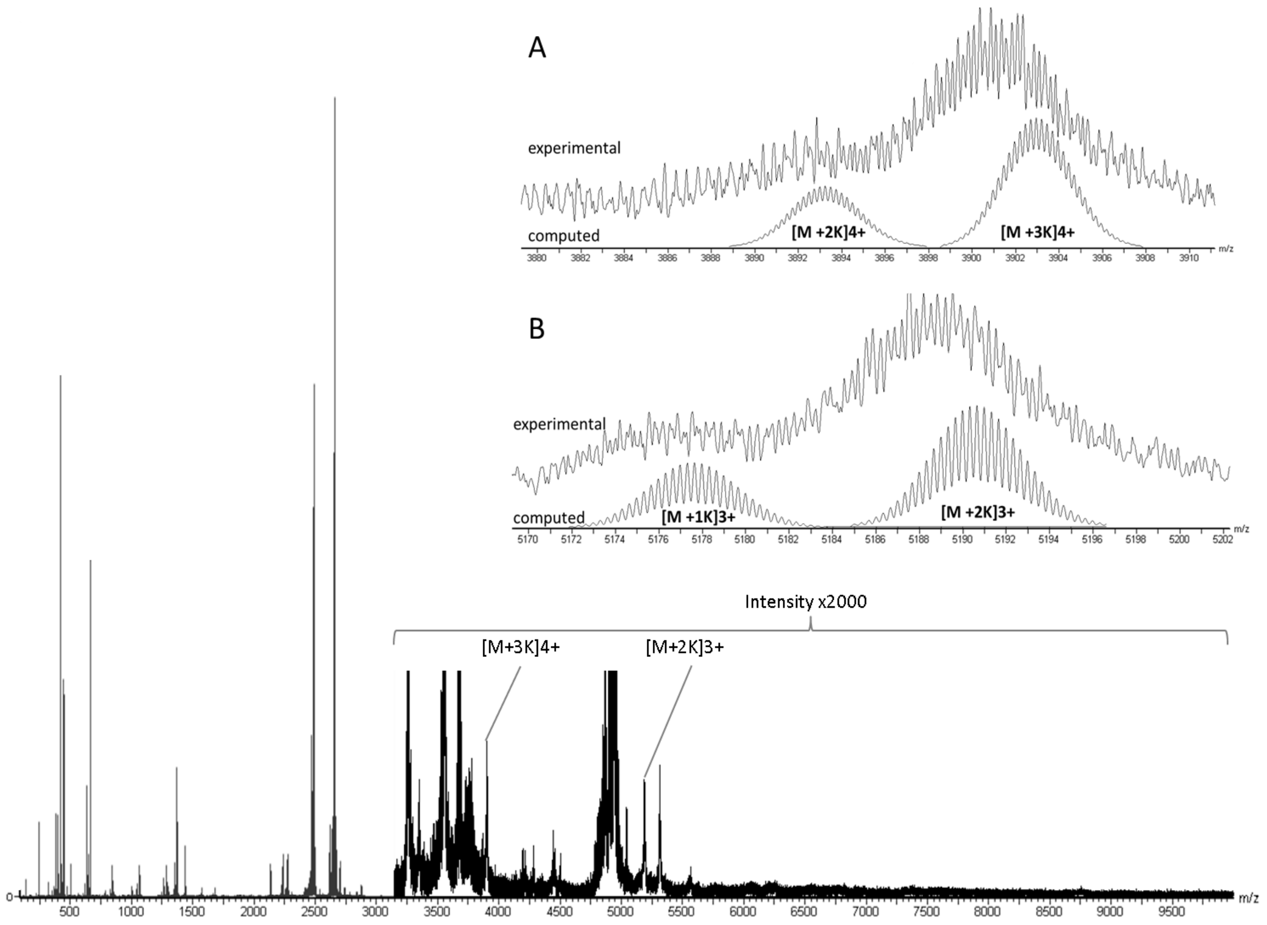

Supplementary Figure 6. Mass spectrum of 7-rotaxane **10** sample. Insets show peaks corresponding to species at 4+ (A) and 4+ (B) charge states presenting as potassium adducts. Computed theoretical isotopic distributions based on the M_w=15493 Da, are included for comparison.

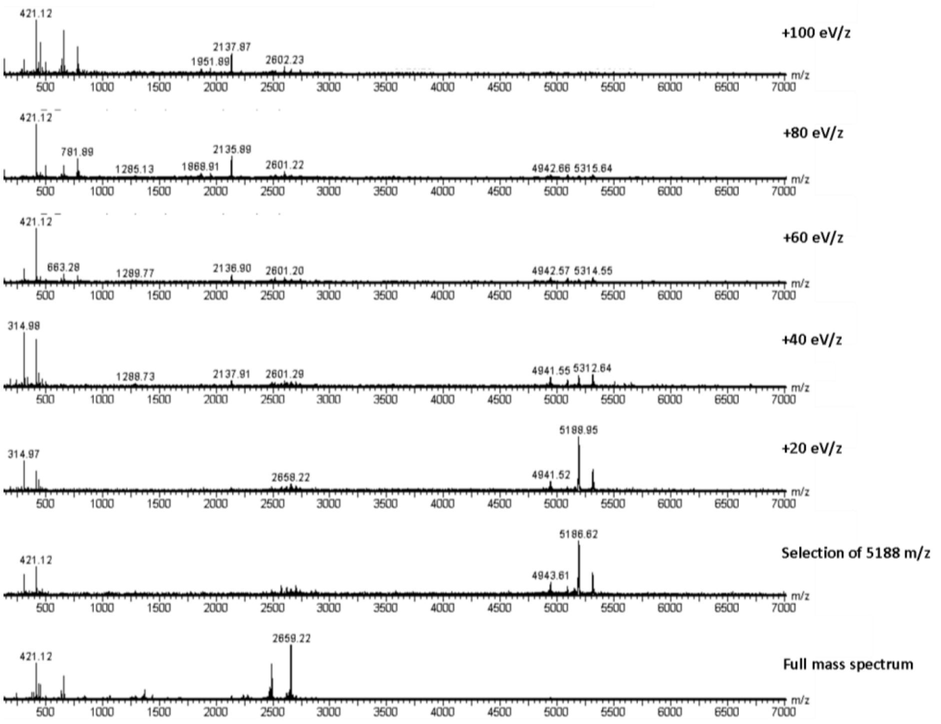

Supplementary Figure 7. Collision induced dissociation of a mass selected 5188 m/z (+3) ion.

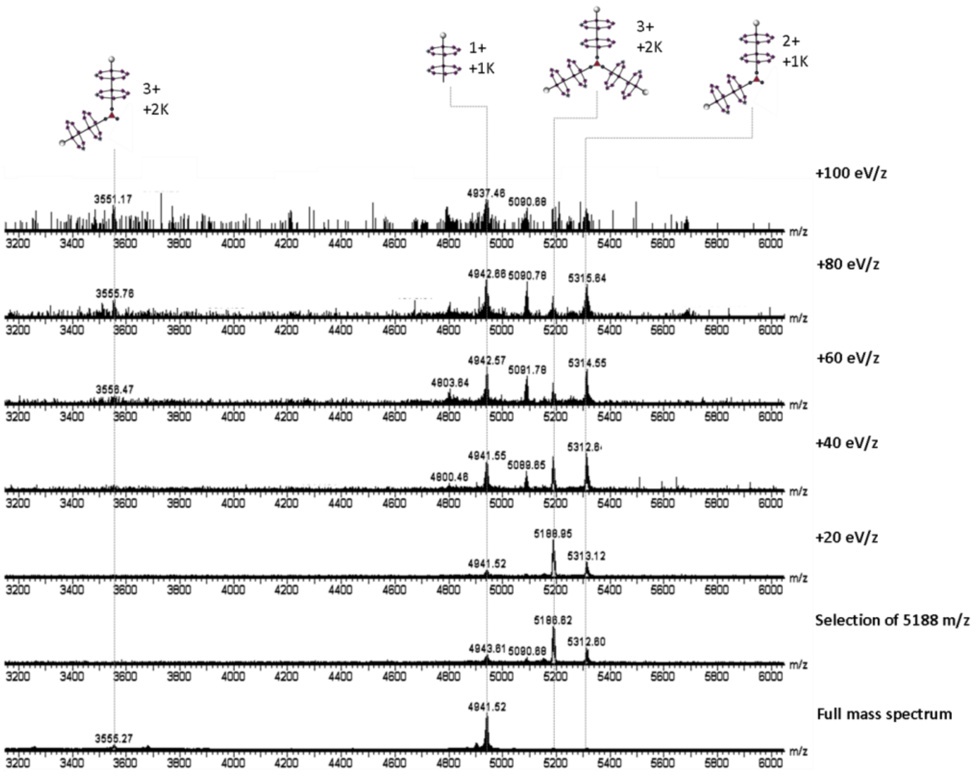

Supplementary Figure 8. Collision induced dissociation of a mass selected 5188 m/z (+3) ion (3000-6000 m/z). Proposed assignments of the intact 7-rotaxane **10** and its fragments.

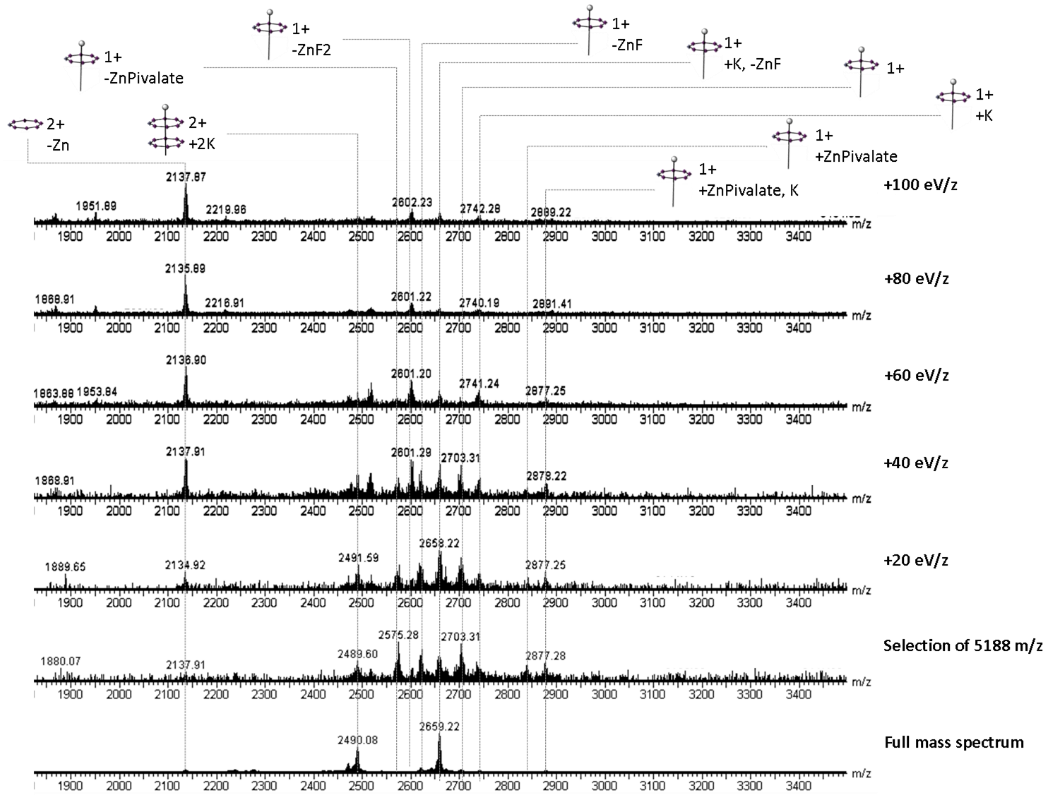

Supplementary Figure 9. Collision induced dissociation of a mass selected 5188 m/z (+3) ion (2000-3500 m/z). Proposed assignments of the fragment ions consisting of a {Cr_7Zn} rings complexed with a thread **C**.

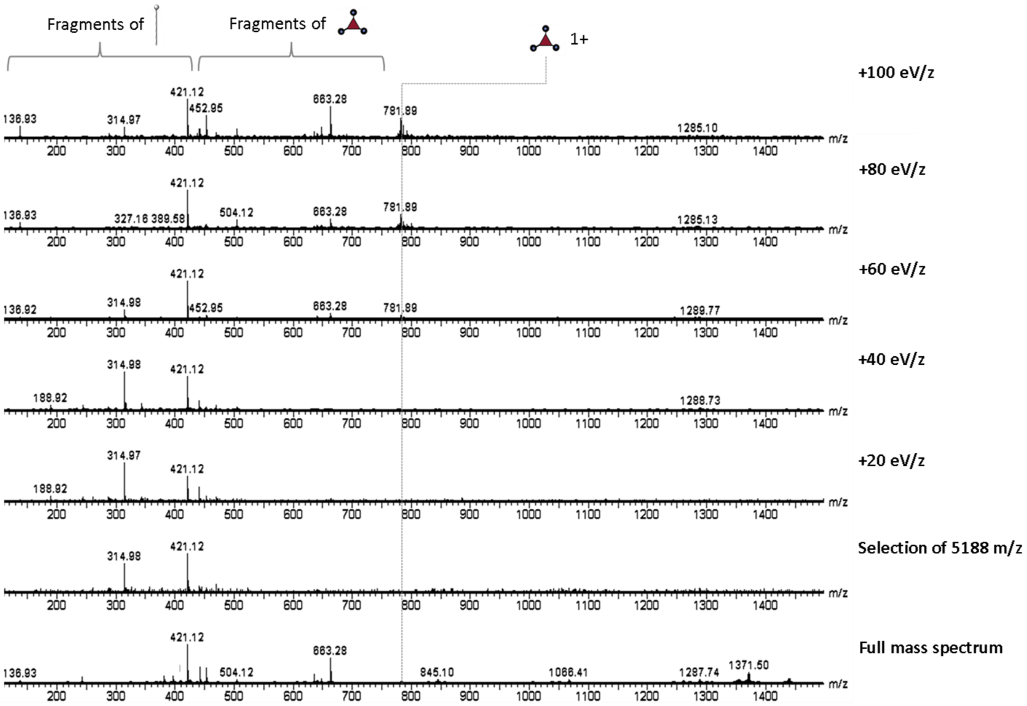

Supplementary Figure 10. Collision induced dissociation of a mass selected 5188 m/z (+3) ion (100-1500 m/z). {Fe_2Co} triangle is seen along with its fragments and fragments of the thread **C.**

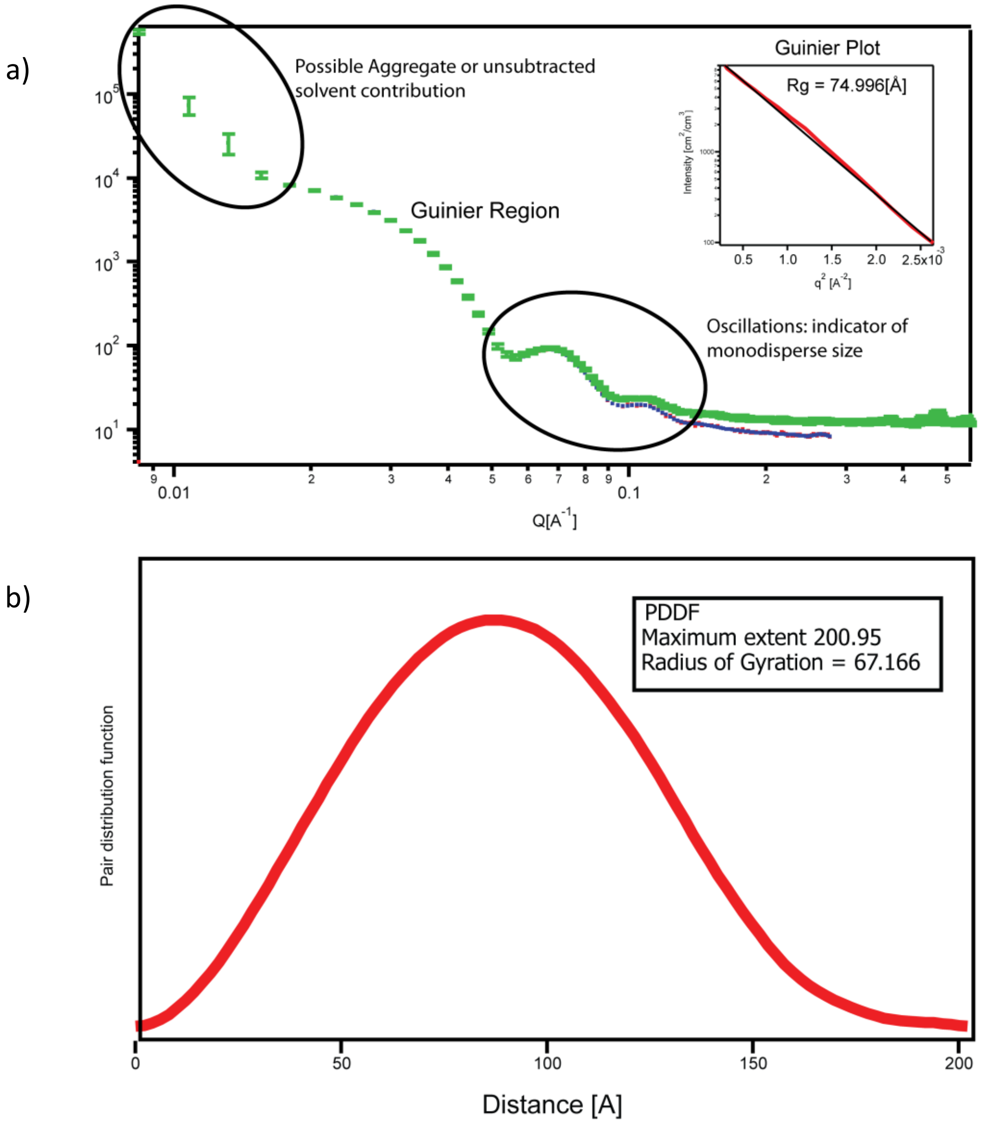

Supplementary Figure 11. Test of Instrument Capability. SAXS data collected on suspended 20 nm gold nanoparticles, as supplied with no dilution, collected on a laboratory SAXS instrument in a 1.6 mm borosilicate capillary. Source Xenocs Micro-focus Cu-K_{α}. Detector – Dectris Pilatus 100. a) Raw data, at low q the Guinier plot is a good fit to a straight line (inset). This region can also be fitted to a Porod plot with q^{-4}, which would be consistent with a smooth surface. b) PDDF data that indicates a maximum consistent with expected size of the particles. Irena routines were used for determining Guinier and PDDF

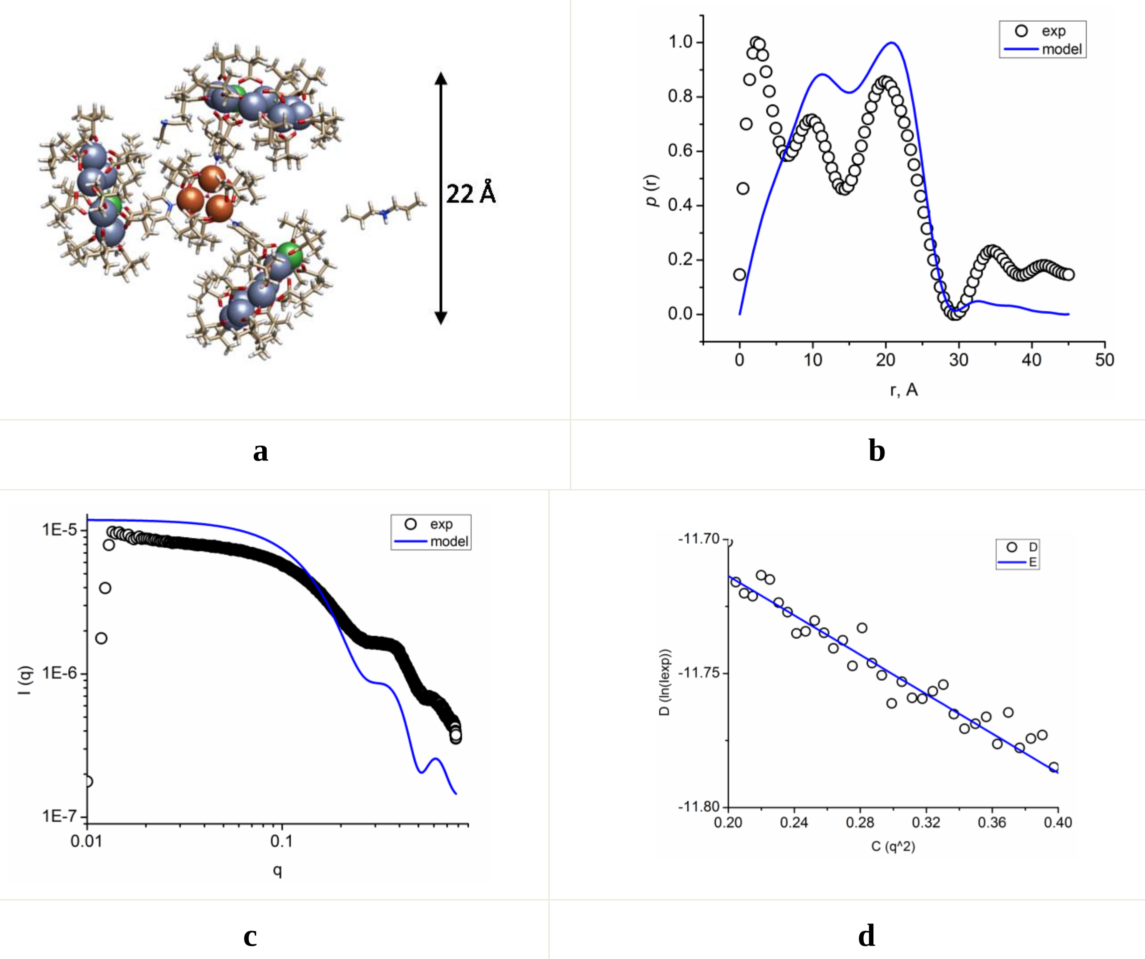

Supplementary Figure 12. Comparison of analysis software. Data collected at Diamond synchrotron. Dilution 5mg/mL in toluene. Data presented has been corrected for toluene contribution. a) The crystal structure of the molecule studied. b) The pair-distance distribution function (PDDF) from raw scattering data features characteristic 12 Å and 22 Å peaks matching those in the model PDDF. c) and d) The R_g obtained using the Guinier equation (Figure 1d, $R_{g\ exp}$ = 10.5 Å) is smaller than that from the simulated PDF ($R_{g\ model}$ = 12.0 Å) (Figure S11c). The latter value is larger than that obtained from the experimental PDF ($R_{g\ exp,\ \mathrm{GNOM}}$ = 10.5 Å). The analysis in supplementary figure 12(b-d) has employed Primus, GNOM and Crysol.

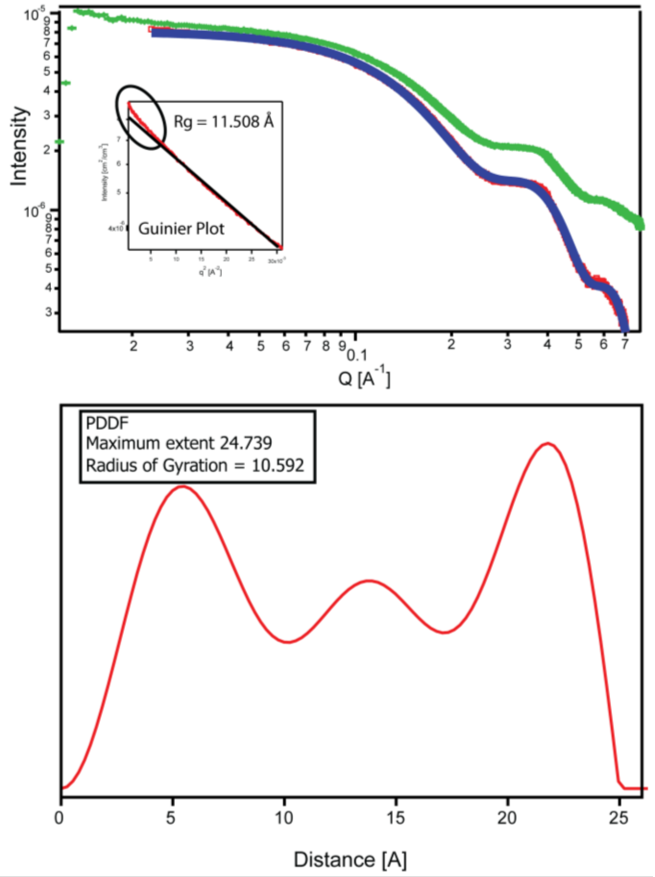

Supplementary Figure 13. Comparison of analysis using Irena routines.[8]

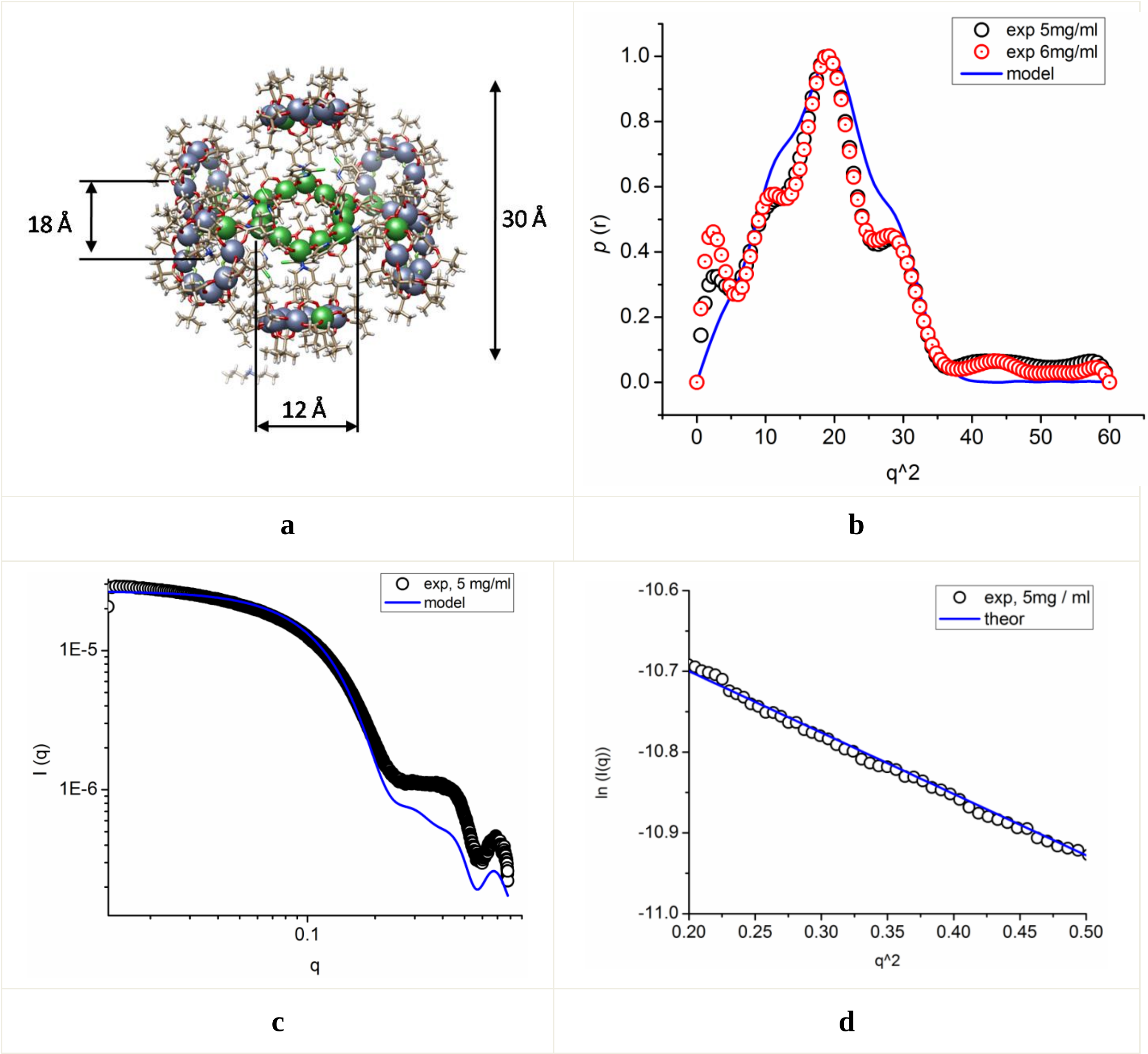

Supplementary Figure 14. Comparison of synchrotron to laboratory SAXS data. a) Crystal structure of the compound studied. b), c) and d). Data collected from Diamond synchrotron for molecule analysed using GNOM, Crysol and. b) also demonstrates that varying concentration has negligible affect.

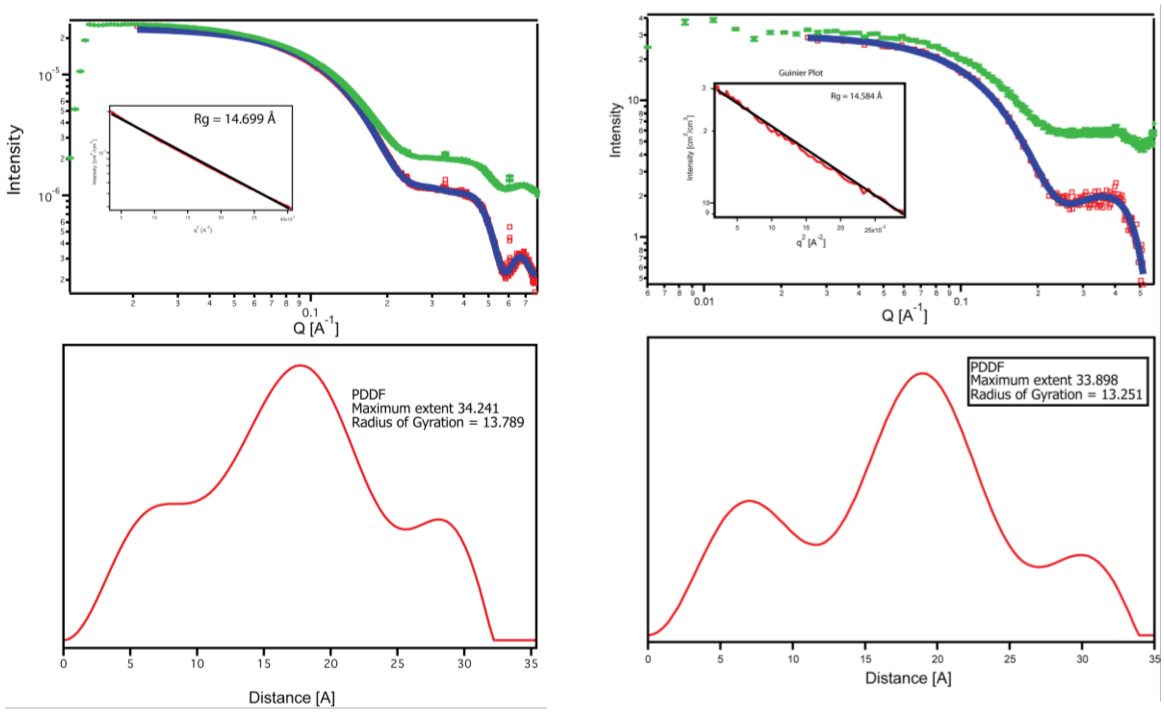

Supplementary Figure 15. Left hand panels show analysis from synchrotron data employing Irena routines within the Igor environment. Right hand panels show the same molecule collected with laboratory instrument and analysed with Irena routines. Top spectra show the fits for PDDF displayed in the bottom panel. The inset in the top panel displays Guinier plot for the data.

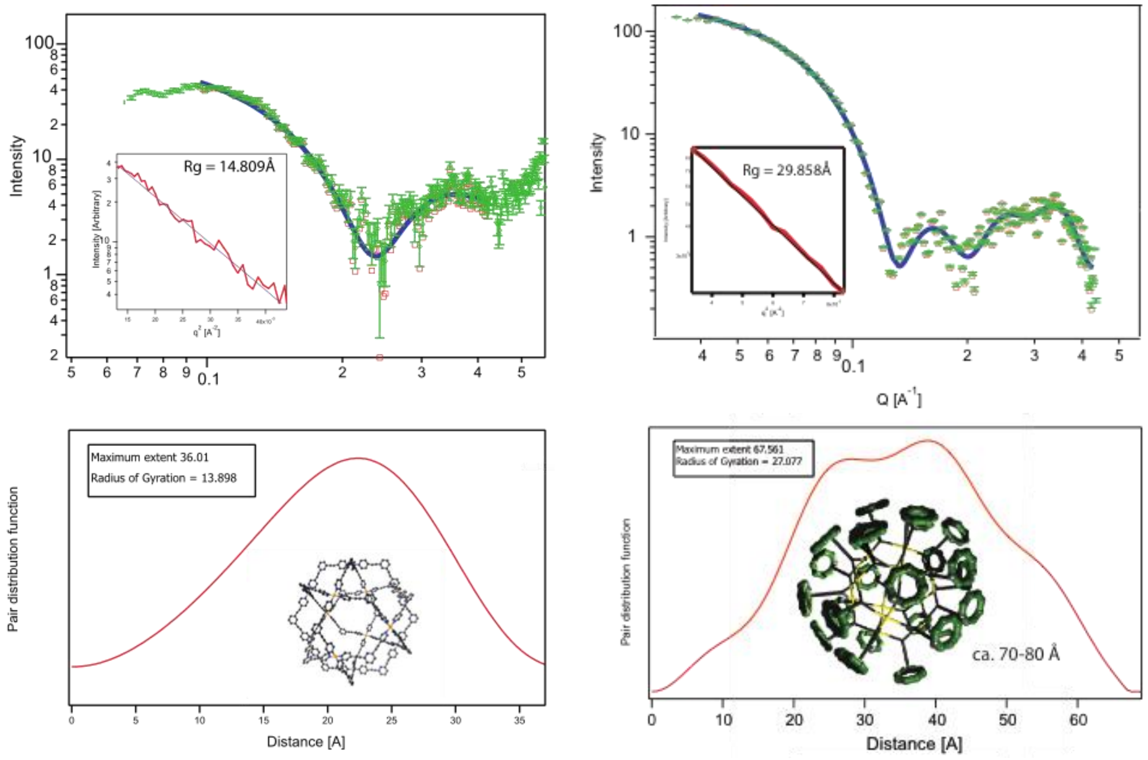

Supplementary Figure 16. Left hand panels are data collected from known Pd_{12} cage from the Fujita group.[13] Right hand panel data collected from unknown synthesised molecule.[12] Top spectra show the fits for PDDF displayed in the bottom panel. The inset in the top panel displays Guinier plot for the data.

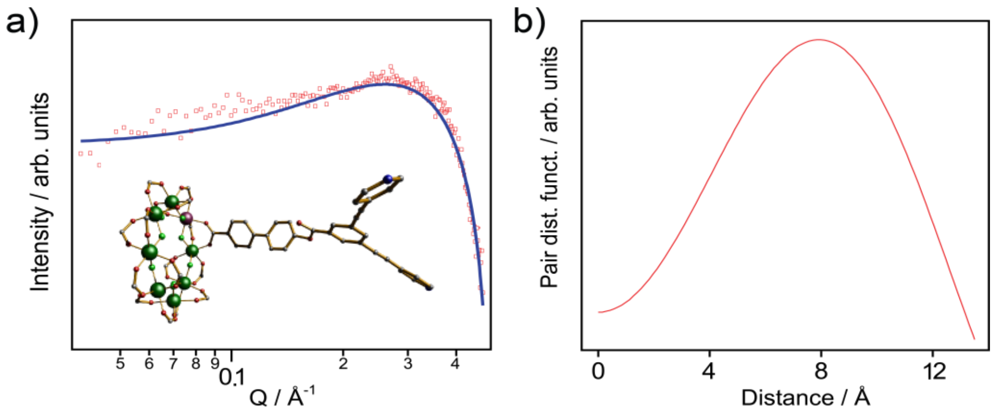

Supplementary Figure 17 a) Experimental SAXS data of compound shown. The solid line is the fit that is associated with the pair distribution function plot shown in b).

Supplementary Tables

Supplementary Table 1. Crystallographic information for 2, 4, 7 and 8.

	[3]-rotaxane (**2**)	[4]-rotaxane (**4**)	[5]-rotaxane (**7**)	[5]-rotaxane (**8**)
Crystal colour	Green	Green	Green	Green
Crystal size (mm)	0.08 × 0.07 × 0.07	0.6 × 0.6 × 0.4	0.3 x 0.2 x 0.1	0.15 × 0.05 × 0.05
Crystal system	Orthorhombic	Triclinic	Triclinic	Triclinic
Space group, *Z*	8	2	1	2
a (Å)	28.9075(3)	25.5047(5)	24.4826(9)	17.4882(5)
b (Å)	29.7016(2)	31.7138(4)	27.807(1)	31.3959(7)
c (Å)	78.0320(9)	31.7614(5)	28.5719(9)	57.060(2)
α (°)	90	73.312(2)	103.388(3)	78.323(2)
β (°)	90	86.822(2)	98.656(3)	84.724(2)
γ (°)	90	78.983(2)	115.074(4)	89.635(2)
V (Å^3)	66998(2)	24154.7(7)	16443(1)	30549(2)
Density (Mg.m^{-3})	1.138	1.144	1.196	1.149
Wavelength (Å)	1.54184	0.6889	1.54184	0.6889
Temperature (K)	100	100	100	100
μ (Mo-Kα) (mm^{-1})	4.419	0.690	4.536	0.709
2Θ range (°)	3.738 to 117.868	3.038 to 51.006	2.618 to 53.507	3.056 to 37.696
Reflns collected	545172	222171	123071	116835
Independent reflns (R_{int})	48103	96021	33412	47292
Reflns used in refinement, *n*	48103	96021	33412	47292
L.S. parameters, *p*	3339	4693	3170	6154
No. of restraints, *r*	696	921	6580	25297
$R1$ $(F)^a$ $I > 2.0\sigma(I)$	0.1164	0.0635	0.0913	0.1727
$wR2(F^2)$,[a] all data	0.3377	0.1999	0.2958	0.2290
$S(F^2)$,[a] all data	1.025	0.995	1.023	1.462

[a] $R1(F) = \Sigma(|F_o| - |F_c|)/\Sigma|F_o|$; [b] $wR^2(F^2) = [\Sigma w(F_o^2 - F_c^2)^2/\Sigma wF_o^4]^{½}$; [c] $S(F^2) = [\Sigma w(F_o^2 - F_c^2)^2/(n + r - p)]$

Supplementary Table 2. Phase relaxation times and two-qubit gate times for **2**, **4**, **7** and **8**.

Compound	T (K)	T_1(ns)	T_{SD} (ns)	T_M (ns)	Stretch parameter, s
**2**	2.6	31696 ± 113	15909 ± 117	789 ± 1	1.670 ± 0.002
**4**	2.6	26327 ± 216	12286 ± 184	704 ± 1	1.610 ± 0.004
**5**	2.6	22030 ± 48	9102 ± 110	797 ± 1	1.711 ± 0.003
**7**	2.6	25792 ± 71	12403 ± 80	798 ± 1	1.688 ± 0.002

Supplementary Methods

Synthetic details

Unless stated otherwise, all reagents and solvents were used without further purification. The syntheses of the hybrid organic-inorganic rotaxanes were carried out in Erlenmeyer Teflon® FEP flasks supplied by Fisher. Column chromatography was carried out using Silica 60A (particle size 35-70 µm, Fisher, UK) as the stationary phase, and TLC was performed on precoated silica gel plates (0.25 mm thick, 60 F254, Merck, Germany) and observed under UV light. NMR spectra were recorded on Bruker AV 400, and Bruker DMX 500 instruments. Chemical shifts are reported in parts per million (ppm) from low to high frequency and referenced to the residual solvent resonance. ESI mass spectrometry, MALDI-TOF spectrometry and microanalysis were carried out by the services at the University of Manchester.

Synthesis of organic threads

2-phenyl-*N*-{[4'-(pyridin-4-yl)-(1,1'-biphenyl)-4-yl]methyl}ethanamine (A):

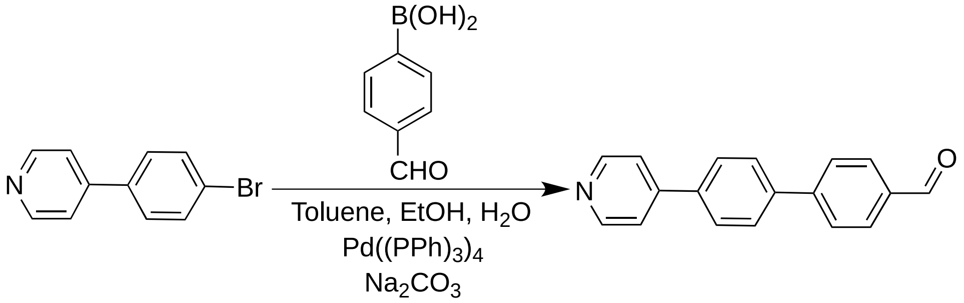

4'-(pyridin-4-yl)-[1,1'-biphenyl]-4-carbaldehyde: To a solution of 4-(4-bromophenyl)pyridine (0.57 g, 2.4 mmol) in 40 mL of toluene, 12 mL of ethanol and 4 mL of water, 4-formylphenylboronic acid (0.4 g, 2.68 mmol) and Na_2CO_3 (1.2 g) were added and the solution was purged with N_2 for 15 min. $Pd(PPh_3)_4$ (10 % molar) was added to the flask and the mixture heated for 18 hours under nitrogen atmosphere. The solvent mixture was evaporated. The solid was redissolved in chloroform (40 mL), washed with water (2x 50 mL) and dried over anhydrous magnesium sulphate and evaporated. A light yellow liquid was obtained in 80 % yield (0.51 g). ESI-MS (sample dissolved in MeOH, run in MeOH): m/z = 260 $[M+H]^+$. ^1H NMR (400 MHz, 293K, $CDCl_3$): δ = 7.2-7.4 (m, 2H), 7.6 (d, 2H), 7.8 (d, 2H), 7.9 (d, 2H), 7.8 (d, 2H), 8.5 (d, 2H), 9.9 (s, 1H). ^{13}C NMR (75 MHz, 293K, $CDCl_3$): δ = 123.2; 123.6; 125.2; 129.8; 130.0; 131.4; 133.6; 142.3; 142.8; 146.1; 150.5; 198.2.

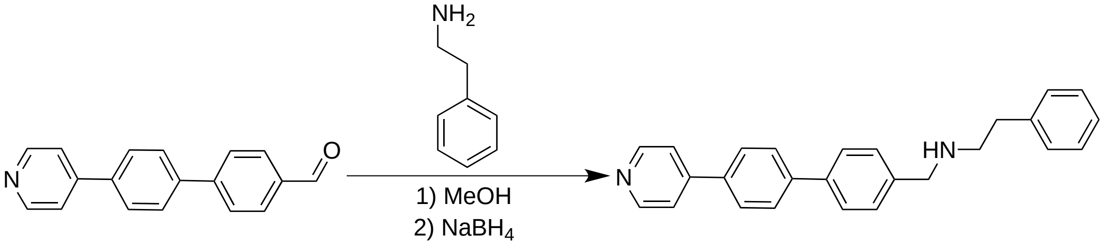

2-phenyl-*N*-{[4'-(pyridin-4-yl)-(1,1'-biphenyl)-4-yl]methyl}ethanamine: To a solution of 4'-(pyridin-4-yl)-[1,1'-biphenyl]-4-carbaldehyde (0.5 g, 1.9 mmol) in 30 mL of methanol, phenylethylamine (0.25 mL, 1.9 mmol) in 5 mL methanol was added and the reaction mixture was refluxed for 3 h under nitrogen atmosphere, allowed to stir at room temperature overnight. $NaBH_4$ (5 equivalents) was added and reaction mixture was stirred during 12 h under nitrogen atmosphere. The reaction was quenched with water and the solvent was evaporated. The solid was redissolved in chloroform (60 mL), washed with water (2x 50 mL) and dried over anhydrous magnesium sulphate and evaporated. A white solid was obtained in 80 % yield (0.55 g). ESI-MS (sample dissolved in MeOH, run in MeOH): m/z = 365 $[M+H]^+$. ^1H NMR (400 MHz, 293K, $CDCl_3$): δ = 2.81-2.93 (m, 4H), 3.89 (s, 2H), 7.2-7.5 (m, 7H), 7.6 (d, 2H), 7.8 (d, 2H), 7.9 (d, 2H), 7.8 (d, 2H), 8.5 (d, 2H). ^{13}C NMR (75 MHz, 293K, $CDCl_3$): δ = 37.8; 46.9; 51.3; 122.8; 125.4; 125.6; 126.0; 127.4; 128.8; 129.7; 129.9; 130.1; 131.5; 133.6; 142.1; 146.4; 151.5; 167.7.

4-phenyl-*N*-(4-(pyridin-4-yl)benzyl)butan-1-amine (B):

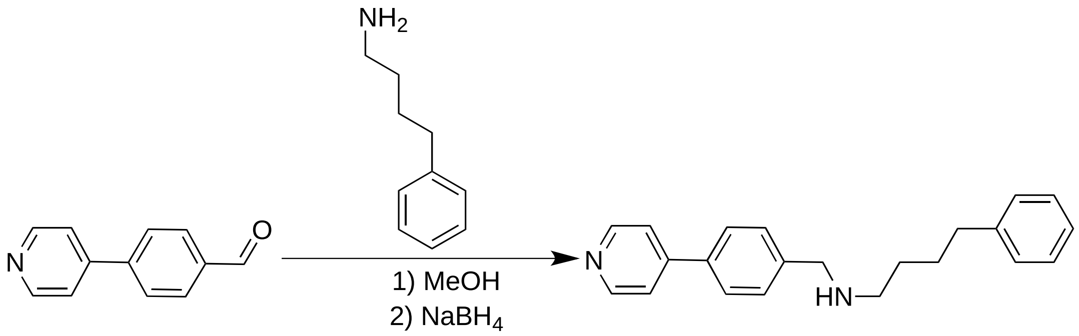

To a solution of 4-pyridine phenylaldehyde (0.84 g, 4.3 mmol) in 30 mL of methanol, phenylbutylamine (0.72 mL, 4.3 mmol) in 5 mL methanol was added and the reaction mixture was refluxed for 3 h under N_2 atmosphere, allowed to stir at room temperature overnight. $NaBH_4$ (5 equivalents) was added and reaction mixture was stirred during 12 h under nitrogen atmosphere. The reaction was quenched with water and the solvent was evaporated. The solid was redissolved in chloroform (60 mL), washed with water (2x 50 mL) and dried over anhydrous magnesium sulphate and evaporated. A light yellow liquid was obtained in 80 % yield (1.4 g). ESI-MS (sample dissolved in MeOH, run in MeOH): m/z = 317 $[M+H]^+$. ^1H NMR (400 MHz, 293K, $CDCl_3$): δ = 1.67-1.90 (m, 4H), 2.81-2.93 (m, 4H), 3.89 (s, 2H), 7.2-7.3 (m, 5H), 7.5 (d, 2H), 7.6 (d 2H) 7.7 (d, 2H), 8.7 (d, 2H). ^{13}C NMR (75 MHz, 293K, $CDCl_3$): δ = 30.5; 31.3; 33.5; 46.3; 53.6; 123.2; 123.6; 125.2; 128.2; 129.0; 129.4; 133.5; 136.9; 142.8; 150.1; 167.7.

N^1-(4-(methylthio)benzyl)-N^{12}-(4-(pyridin-4-yl)benzyl)dodecane-1,12-diamine (C):

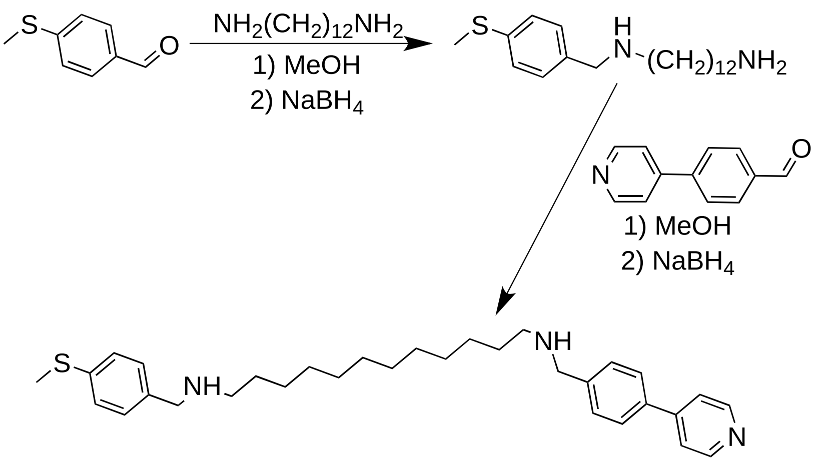

To a solution of 1,12-diaminododecane (0.7 g, 3.5 mmol) in 30 mL methanol, 4-methylthiobenzaldehyde (0.47 mL, 3.5 mmol) in 5 mL methanol was added and the reaction mixture was refluxed for 3 h under nitrogen atmosphere and then cooling to room temperature. $NaBH_4$ was added and reaction mixture stirred over night under N_2 atmosphere. The reaction was quenched with water and the solvent mixture was evaporated. The solid was redissolved in chloroform (60 mL), washed with water (2x 60 mL), dried over anhydrous magnesium sulphate and evaporated. To a solution of the amine in 30 mL methanol, 4-pyridine phenylaldehyde (0.64 mL, 3.5 mmol) in 5 mL methanol was added and the reaction mixture was refluxed for 3 hr under nitrogen atmosphere, allowed to stir at room temperature overnight. $NaBH_4$ was added and reaction mixture stirred over night under nitrogen atmosphere. The reaction was quenched with water and evaporated. The solid was redissolved in chloroform (50 mL), washed with water (2x 50 mL) and dried over anhydrous magnesium sulphate and evaporated (60 % yield). The product was recrystallized from methanol and confirmed by spectroscopic analysis. ES-MS (sample dissolved in dichloromethane, run in MeOH): m/z = 504 $[M+H]^+$. ^1H NMR (400 MHz, 293K, $CDCl_3$): δ = 1.2-1.6 (m, 20H), 2.3 (s, 3H), 2.4-2.6 (m, 4H), 3.7 (s, 2H), 3.8 (s, 2H), 7.1-7.3 (m, 4 H), 7.3-7.5 (m, 4 H), 7.6 (d, 2H), 8.6 (d, 2H). ^{13}C NMR (75 MHz, 293K, $CDCl_3$): δ = 18.1; 27.1; 27.6; 27.9; 28.1; 28.4; 28.8; 29.0; 29.3; 29.6; 30.1; 30.7; 33.5; 49.4; 52.7; 123.6; 125.3; 128.5; 129.0; 129.1; 133.3; 136.3; 142.4; 150.3; 150.8; 166.0.

N^1-(4-(methylthio)benzyl)-N^{12}-(pyridin-4-ylmethyl)dodecane-1,12-diamine (D):

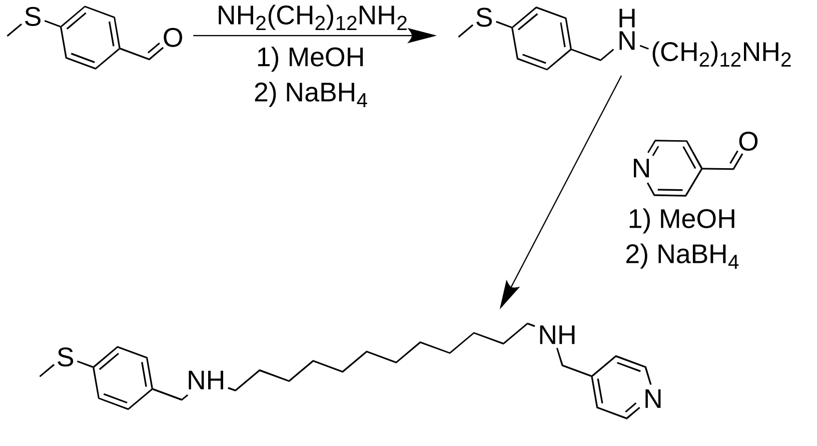

To a solution of 1,12-diaminododecane (2 g, 10 mmol) in 30 mL methanol, 4-methylthiobenzaldehyde (1.35 mL, 10 mmol) in 5 mL methanol was added and the reaction mixture was refluxed for 3 h under nitrogen atmosphere and then cooling to room temperature. The imine precipitated formed was filtered and washed with MeOH. To a solution of the imine in 30 mL methanol, 4-pyridinecarboxaldehyde (0.95 mL, 10 mmol) in 5 mL methanol was added and the reaction mixture was refluxed for 3 h under nitrogen atmosphere, allowed to stir at room temperature overnight. $NaBH_4$ was added and reaction mixture stirred over night under nitrogen atmosphere. The reaction was quenched with water and evaporated. The solid was redissolved in chloroform (60 mL), washed with water (2x 50 mL) and dried over anhydrous magnesium sulphate and evaporated (60 % yield). The product was confirmed by spectroscopic analysis. ES-MS (sample dissolved in dichloromethane, run in MeOH): m/z = 428 $[M+H]^+$. ^1H NMR (400 MHz, 293K, $CDCl_3$): δ = 1.3-1.6(m, 20H), 2.4(s, 3H), 2.70 (t, 2H), 2.80 (t, 2H), 3.60 (s, 2H), 3.70 (s, 2H), 7.09-7.3(Ph+py, 7 Hs), 8.4(d, py, 2H). ^{13}C NMR (75 MHz, 293K, $CDCl_3$): δ = 18.1; 27.1; 27.2; 27.3; 27.9; 28.1; 28.4; 28.8; 29.0; 29.6; 30.1; 30.7; 33.5; 49.4; 52.7; 128.5; 133.3; 136.3; 142.4; 150.3; 150.8; 166.0.

Synthesis of [2]- and [3]-rotaxanes

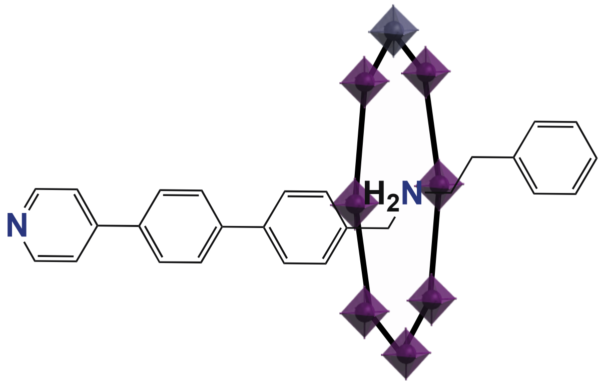

$[AH_2\{Cr_7Ni(\mu\text{-}F)_8(O_2C^tBu)_{16}\}]$ (1): Pivalic acid (20.0 g, 195 mmol), 2-phenyl-*N*-{[4'-(pyridin-4-yl)-(1,1'-biphenyl)-4-yl]methyl}ethanamine (0.6 g, 1.6 mmol), and $CrF_3\cdot 4H_2O$ (2 g, 11 mmol) were heated at 140°C with stirring in a Teflon flask for 0.5 h, then $[Ni_2(H_2O)(O_2CCMe_2)_4(HO_2CCMe_2)_4]$ (1.11 g, 2.4 mmol) was added. After 1 h the temperature of the reaction was increased to 160°C for 24 h. The flask was cooled to room temperature, and then acetonitrile (35 mL) was added while stirring. The green microcrystalline product was collected by filtration, washed with a large quantity of acetonitrile (200 mL), dried in air, and then extracted with toluene (250 mL). Flash chromatography (toluene and then ethyl acetate/toluene (3/7)) afforded the desired [2]-rotaxane as a green crystalline solid (0.6 g) in 14 % yield. Elemental analysis (%) calcd for $C_{108}H_{172}Cr_7F_8N_2NiO_{32}$: Cr 14.08, Ni 2.27, C 50.18, H 6.71, N 1.08; found: Cr 14.53, Ni 2.11, C 49.90, H 6.29, N 1.19. ESI-MS (sample dissolved in THF, run in MeOH): m/z = 2559 $[M+H]^+$; 2581 $[M+Na]^+$.

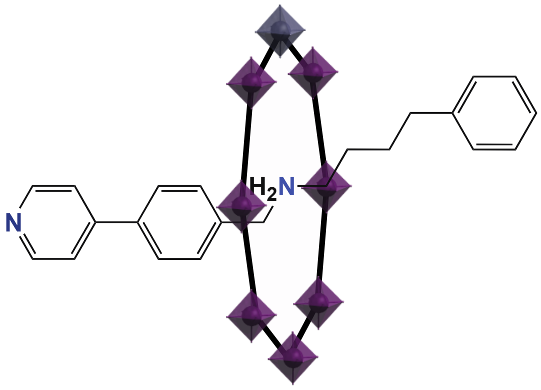

[$BH_2\{Cr_7Ni(\mu\text{-}F)_8(O_2C^tBu)_{16}\}$] (3): Pivalic acid (20.0 g, 195 mmol), *N*-[(1,1'-biphenyl)-4-ylmethyl]-4-phenylbutan-1-amine (0.5 g, 2.4 mmol), and $CrF_3{\cdot}4H_2O$ (2.2 g, 15 mmol) were heated at 140°C with stirring in a Teflon flask for 0.5 h, then [$Ni_2(H_2O)(O_2CCMe_2)_4(HO_2CCMe_2)_4$] (0.98 g, 4.8 mmol) was added. After 1 h the temperature of the reaction was increased to 160°C for 24 h and the reaction was carried out under N_2 atmosphere. The flask was cooled to room temperature, and then acetonitrile (35 mL) was added while stirring. The green microcrystalline product was collected by filtration, washed with a large quantity of acetonitrile (200 mL), dried in air, and then extracted with toluene (300 mL). Flash chromatography (toluene and then ethyl acetate/toluene (3/7)) afforded the desired [2]-rotaxane as a green crystalline solid (0.6 g) in 10 % yield. Elemental analysis (%) calcd for $C_{102}H_{168}Cr_7F_8N_2NiO_{32}$: Cr 14.50, Ni 2.33, C 48.80, H 6.78, N 1.11; found: Cr 14.43, Ni 2.31, C 48.61, H 6.62, N 1.14. ESI-MS (sample dissolved in THF, run in MeOH): m/z = 2511 $[M+H]^+$; 2532 $[M+Na]^+$.

[$BH_2\{Cr_7Zn(\mu\text{-}F)_8(O_2C^tBu)_{16}\}$] (3b): The compound **3b** was prepared by an analogous procedure to that for **3** by using $ZnCO_3$ (1.6 g, 4.8 mmol) instead of [$Ni_2(H_2O)(O_2CCMe_2)_4(HO_2CCMe_2)_4$] (1.21 g) in 20 % yield. Elemental analysis (%) calcd for $C_{102}H_{168}Cr_7F_8N_2ZnO_{32}$: Cr 14.47, Zn 2.60, C 48.70, H 6.73, N 1.11; found: Cr 14.42, Zn 2.41, C 48.71, H 6.69, N 1.14. ESI-MS (sample dissolved in THF, run in MeOH): m/z = 2518 $[M+H]^+$; 2539 $[M+Na]^+$.

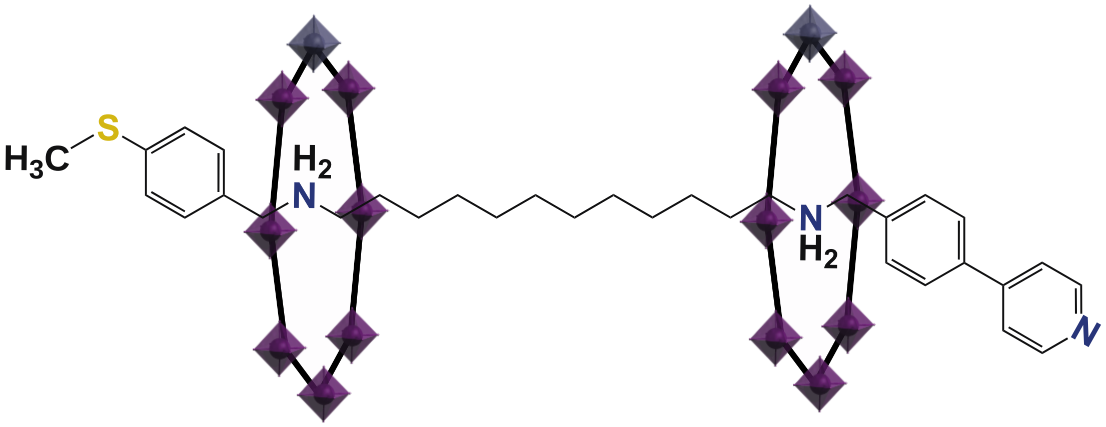

[$CH_2\{Cr_7Ni(\mu\text{-}F)_8(O_2C^tBu)_{16}\}_2$] (5): Pivalic acid (24 g, 230 mmol), N^1-(4-(methylthio)benzyl)-N^{12}-(4-(pyridin-4-yl)benzyl)dodecane-1,12-diamine (0.47 g, 0.94 mmol), $CrF_3{\cdot}4H_2O$ (2.36 g, 13 mmol) were heated at 140°C with stirring in a Teflon flask for 0.5 h, then [$Ni_2(H_2O)(O_2CCMe_2)_4(HO_2CCMe_2)_4$] (1.5 g, 4.47 mmol) was

added. After 1 h the temperature of the reaction was increased to 160°C for 24 h and the reaction was carried out under N_2 atmosphere. The flask was cooled to room temperature, and then acetonitrile (35 mL) was added while stirring. The green microcrystalline product was collected by filtration, washed with a large quantity of acetonitrile (200 mL), dried in air, and then extracted with toluene (250 mL). Flash chromatography (toluene then toluene/ethyl acetate 7/3) afforded the desired [3]-rotaxane as a green crystalline solid (1.1 g) in 23 % yield. Elemental analysis (%) calcd for $C_{192}H_{335}Cr_{14}F_{16}N_3Ni_2O_{64}S_1$: Cr 14.84, Ni 2.40, C 47.02, H 7.37, N 0.85; found: Cr 13.92, Ni 2.39, C 47.71, H 6.88, N 0.77.

[$CH_2\{Cr_7Zn(\mu\text{-}F)_8(O_2C^tBu)_{16}\}_2$] (5b): The compound **5b** was prepared by an analogous procedure to that for **5** by using $ZnCO_3$ (1.5 g, 4.47 mmol) instead of [$Ni_2(H_2O)(O_2CCMe_2)_4(HO_2CCMe_2)_4$] (1.6 g) in 33 % yield. Elemental analysis (%) calcd for $C_{192}H_{335}Cr_{14}F_{16}N_3Zn_2O_{64}S_1$: Cr 14.84, Zn 2.66, C 47.02, H 7.37, N 0.85; found: Cr 13.98, Zn 2.45, C 47.31, H 7.68, N 0.87.

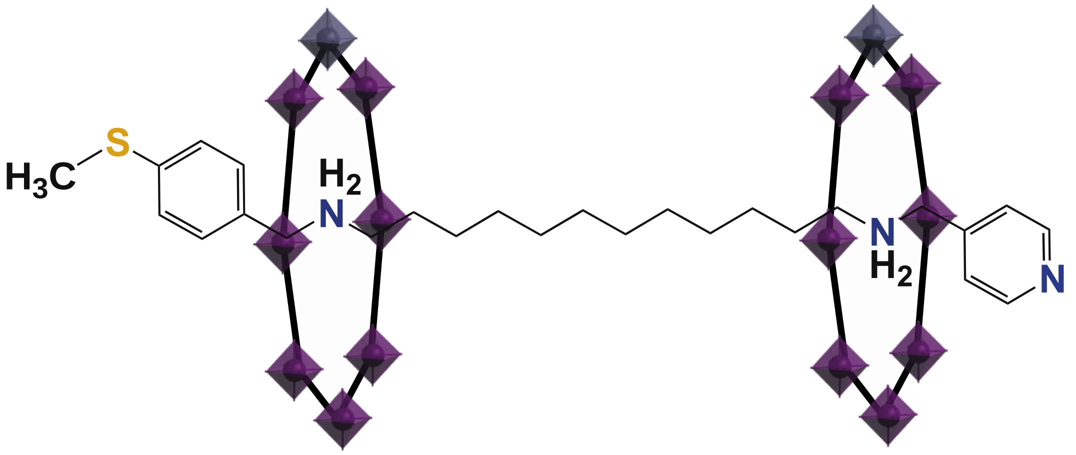

[$DH_2\{Cr_7Ni(\mu\text{-}F)_8(O_2C^tBu)_{16}\}_2$] (6): Pivalic acid (20.0 g, 195 mmol), N^1-(4-(methylthio)benzyl)-N^{12}-(pyridin-4-ylmethyl)dodecane-1,12-diamine (0.67 g, 1.5 mmol), and $CrF_3\cdot4H_2O$ (3.9 g, 21 mmol) were heated at 140°C with stirring in a Teflon flask for 0.5 h, then [$Ni_2(H_2O)(O_2CCMe_2)_4(HO_2CCMe_2)_4$] (2.1 g, 4.47 mmol) was added. After 1 h the temperature of the reaction was increased to 160°C for 24 h. The flask was cooled to room temperature, and then acetonitrile (35 mL) was added while stirring. The green microcrystalline product was collected by filtration, washed with a large quantity of acetonitrile (200 mL), dried in air, and then extracted with hexane (300 mL). Flash chromatography (toluene then toluene/ethyl acetate 9/1) afforded the desired [3rotaxane as a green crystalline solid (1.1 g) in 20 % yield. Elemental analysis (%) calcd for $C_{186}H_{331}Cr_{14}F_{16}N_3Ni_2O_{64}S_1$: Cr 15.12, Ni 2.44, C 46.42, H 6.89, N 0.87, S 0.67; found: Cr 14.57, Ni 2.23, C 46.97, H 7.43, N 0.84, S 0.63.

Synthesis of supramolecular assemblies

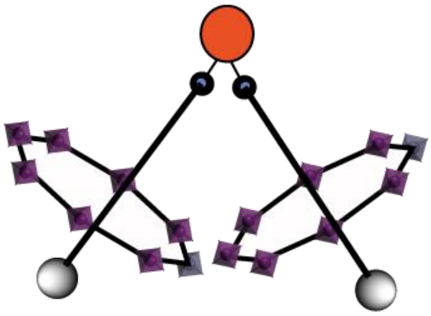

$\{[AH_2\{Cr_7Ni(\mu\text{-}F)_8(O_2C^tBu)_{16}\}]_2[Cu(hfacac)_2]\}$ 2: $[Cu(hfacac)_2]$ (0.01 g, 0.004 mmol) was added to a solution of **1** (0.1 g, 0.008 mmol) in hot acetone (10 mL), and the mixture was refluxed for 5 min. The solution was cooled to room temperature and left to crystallize. Yield: 0.03g (30 %). Elemental analysis (%) calcd for $C_{222}H_{340}Cr_{14}F_{28}N_4Ni_2O_{68}Cu$: Cr 13.01, Ni 2.09, Cu 1.13, C 47.66, H 6.12, N 1.00; found: Cr 12.41, Ni 1.99, Cu 1.47, C 47.09, H 6.64, N 0.96. X-ray quality crystals were obtained for **2** by slow evaporation of acetone solution.

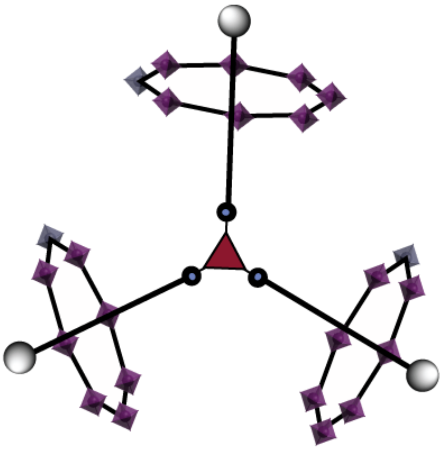

$\{[BH_2\{Cr_7Ni(\mu\text{-}F)_8(O_2C^tBu)_{16}\}]_3[Fe_2Co(\mu_3\text{-}O)(O_2C^tBu)_6]\}$ 4: (0.013 g, 0.01 mmol) was added to a solution of **3** (0.09 g, 0.035 mmol) in hot acetone (10 mL), and the mixture was refluxed for 5 min. and then stirred at r.t. for 4h. Yield: 0.06g (70 %). Elemental analysis (%) calcd for $C_{335}H_{556}Cr_{21}F_{24}N_6Ni_3O_{110}CoFe_2$: Cr 13.12, Ni 2.11, Co 0.70, Fe 1.34, C 48.34, H 6.73, N 1.01; found: Cr 12.78, Ni 2.06, Co 0.55, Fe 1.36, C 48.57, H 7.08, N 0.98. X-ray quality crystals were obtained for **4** by recrystallization from acetone.

$\{[BH_2\{Cr_7Zn(\mu\text{-}F)_8(O_2C^tBu)_{16}\}]_3[Fe_2Co(\mu_3\text{-}O)(O_2C^tBu)_6]\}$ 4b: The compound **4b** was prepared by an analogous procedure to that for **4** by using **3b** as precursor instead of **3**. Yield: 0.07g (70 %). Elemental analysis (%) calcd for $C_{335}H_{556}Cr_{21}F_{24}N_6Zn_3O_{110}CoFe_2$: Cr 13.08, Zn 2.35, Co 0.71, Fe 1.34, C 48.23, H 6.72, N 1.01; found: Cr 12.98, Zn 2.36, Co 0.68, Fe 1.35, C 48.37, H 6.78, N 1.08. MALDI-TOF (sample dissolved in Acetone, run in MeOH): m/z = 2797 $[M+H+Na]^{3+}$.

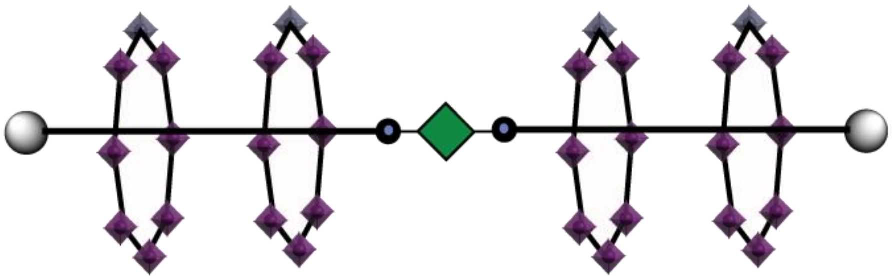

$\{[DH_2\{Cr_7Ni(\mu\text{-}F)_8(O_2C^tBu)_{16}\}_2]_2[Cu_2(O_2C^tBu)_4]\}$ 7: (0.008 g, 0.001 mmol) was added to a solution of **6** (0.1 g, 0.002 mmol) in hot toluene (10 mL), and the mixture was refluxed for 30 min. The solution was cooled to room temperature and left to crystallize. Yield: 0.07 g (70 %)**.** Elemental analysis (%) calcd for $C_{392}H_{698}Cr_{28}F_{32}N_6Ni_4O_{136}S_2Cu_2$: Cr 14.32, Ni 2.31, Cu 1.21, C 46.33, H 6.92, N 0.82; found: Cr 12.04, Ni 1.96, Cu 1.23, C 47.01, H 7.09, N 0.74. X-ray quality crystals were obtained for **7** by recrystallization from slow evaporation of toluene.

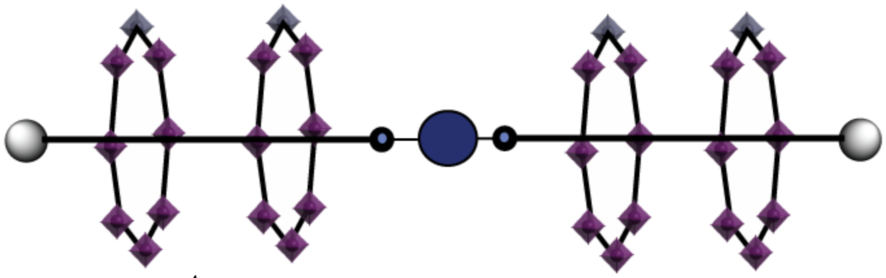

$\{[CH_2\{Cr_7Ni(\mu\text{-}F)_8(O_2C^tBu)_{16}\}_2]_2[Cu(NO_3)_2]\}$ 8: $Cu(NO_3)_2{\cdot}6H_2O$ (0.0018 g, 0.001 mmol) was added to a solution of **5** (0.1 g, 0.002 mmol) in hot toluene (10 mL), and the mixture was refluxed for 30 min. The solution was cooled to room temperature and left to crystallize. Yield: 0.07 g (70 %)**.** Elemental analysis (%) calcd for $C_{392}H_{698}Cr_{28}F_{32}N_6Ni_4O_{136}S_2Cu_2$: Cr 14.32, Ni 2.31, Cu 1.21, C 46.33, H 6.92, N 0.82; found: Cr 12.04, Ni 1.96, Cu 1.23, C 47.01, H 7.09, N 0.74. X-ray quality crystals were obtained for **6** by recrystallization from slow evaporation of toluene.

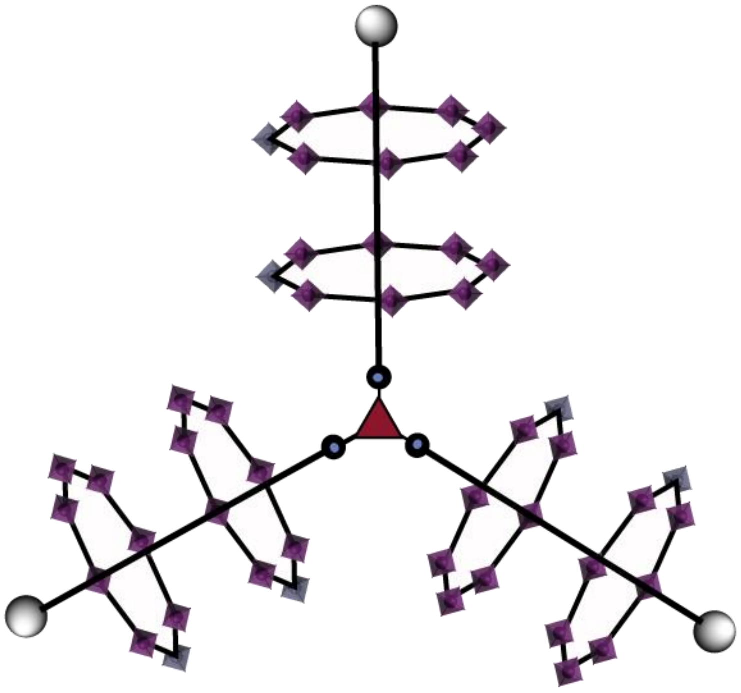

$\{[CH_2\{Cr_7Ni(\mu\text{-}F)_8(O_2C^tBu)_{16}\}_2]_3[Fe_2Co(\mu_3\text{-}O)(O_2C^tBu)_6]\}$ 9: $[Fe_2Co(\mu_3\text{-}O)(O_2C^tBu)_6(H_2O)_3]$ (0.14 g, 2.86 mmol) was added to a solution of **5** (0.01 g, 0.95 mmol) in hot acetone (10 mL), and the mixture was refluxed for 5 min. and then stirred at r.t. for 4 h. Yield: 0.05g (20 %). Elemental analysis (%) calcd for $C_{606}H_{1059}Cr_{42}F_{48}N_9Ni_6O_{205}CoS_3Fe_2$: Cr 14.0, Ni 2.28, Co 0.38, Fe 0.79, C 46.94, H 6.88, N 0.81; found: Cr 12.76, Ni 2.02, Co 0.48, Fe 0.91, C 47.23, H 7.93, N 0.82.

$\{[CH_2\{Cr_7Zn(\mu\text{-}F)_8(O_2C^tBu)_{16}\}_2]_3[Fe_2Co(\mu_3\text{-}O)(O_2C^tBu)_6]\}$ 10: The compound **10** was prepared by an analogous procedure to that for **9** by using **5b** as precursor instead of **5**. Yield: 0.06g (25 %). Elemental analysis (%) calcd for $C_{606}H_{1059}Cr_{42}F_{48}N_9Zn_6O_{205}CoS_3Fe_2$: Cr 14.08, Zn 2.53, Co 0.38, Fe 0.72, C 46.94, H 6.88, N 0.81; found: Cr 14.76, Zn 2.32, Co 0.48, Fe 0.81, C 46.23, H 6.93, N 0.83. ESI-MS (sample dissolved in dicholormethane:acetone): m/z = 5176 $[M+2H+K]^{3+}$; 5188 $[M+H+2K]^{3+}$.

Crystallography

Synchrotron X-ray data were collected at beamline I19 (λ = 0.6889 Å) Diamond Light Source,[1] for [4]-rotaxane (**4**) and [5]-rotaxane (**8**) at temperature of 100 K. Data were measured using CrystalClear-SM Expert 2.0 r5 suite of programs. The data of [3]-rotaxane (**2**) and [5]-rotaxane (**7**) were collected at a temperature of 100 K using a Bruker X8 Prospector diffractometer with Cu-Kα radiation (*λ* = 1.54184 Å), equipped with an Oxford Cryosystems Cobra nitrogen flow gas system. Data were measured using Bruker APEX2 suite of programs. Final crystal and refinement parameters are give in Supplementary Table 1.

X-ray data were processed and reduced using CrysAlisPro suite of programs. Absorption correction was performed using empirical methods (SADABS) based upon symmetry-equivalent reflections combined with measurements at different azimuthal angles.[2-3] All crystal structures were solved and refined against all F^2 values using the SHELXTL suite of programs.[4] Non-hydrogen atoms were refined anisotropically where possible, whereas hydrogen atoms were placed in calculated positions, refined using idealized geometries (riding model) and assigned fixed isotropic displacement parameters. In many of the structures determinations, pivalates ligands are described as disordered over two orientations, with carbon atoms modelled using SADI and SAME bond distance restrain commands, except for compounds **7** and **8** in which the disorder remained unsolved. As well, in crystal structure **2**, **4** and **8**, the threads are described as disordered over two or three orientations, with carbon-carbon and carbon-nitrogen bonds and angles restrained using DFIX and SADI commands. Solvent molecules as acetone (for compounds **2** and **4**) and toluene (for compounds **7** and **8**) were modelled to have idealized geometries using DFIX, FLAT, AFIX and SADI bond distance restrain commands. The atomic displacement parameters (adp) of the pivalate ligands, the thread and the solvent molecules have been restrained using RIGU, SIMU and ISOR commands.

In all the structures, nickel and chromium atoms were described as disordered at metal positions of the ring macrocycle, with the atomic occupancies set to 7/8 for chromium and 1/8 for nickel atoms. Atomic coordinates and displacement parameters were restrained to be equal using EXYZ and EADP commands.

Compounds **2** and **4** present large voids filled with a lot of scattered electron density, the SQUEEZE protocol inside PLATON suites was used to account the void electron density.[5] A large number of A and B alerts were found due to poor crystal quality and data for compound **8**. Approximately all of these alerts (both A and B level) result from the large and deformed/ill-balanced thermal movement of the pivalate ligands and toluene molecules. The cause of this effect can be attributed to the unsolved disorder of these moieties. CCDC 1037544-1037547 contain the supplementary crystallographic data for this paper. These data can be obtained free of charge via www.ccdc.cam.ac.uk/conts/retrieving.html (or from the Cambridge Crystallographic Data Centre, 12 Union Road, Cambridge CB21EZ, UK; fax: (+44)1223-336-033; or deposit@ccdc.cam.ac.uk).

Electron Paramagnetic Resonance Spectroscopy

Pulsed and continous-wave (CW) electron paramagnetic resonance (EPR) measurements were performed at low-temperatures on a a Bruker ElexSys E580 spectrometer operating at X-band frequency (ca. 9.6 GHz). In all cases, dilute (0.0002 M) toluene solutions of compounds were used.

Field sweep spectroscopy

Continous wave EPR spectra for **2**, **4**, **5** and **7** were recorded at X-band (9.67 GHz) and either 2.6 K or 5 K. In all cases, a very distinctive EPR signal with $g_{\perp} = 1.778$ and $g_{II} = 1.720$ was observed , being due to the $S = 1/2$ ground state of the $\{Cr_7Ni\}$ qubit.[6] Echo field-swept EPR data were recorded at 2.6 K with a primary echo sequence, π/2-τ-π-τ-*echo*, with π=32 ns and τ = 300 ns. The maximum resonance intensity is observed at the field position associated to $g_{\perp} = 1.778$ (ca. 3912 G; Supplementary Figure 1).

Inversion recovery

The inversion recovery pulse sequence used was π-t-π/2-τ-π-τ-echo, with π=32 ns, τ = 320 ns and variable *t*. The spin-lattice relaxation time constant, T_1, was deducted by fitting the resulting signal to eq. (1):

$$I(t) = I_1 \exp(-t/T_1) + I_{SD} \exp(-t/T_{SD}) \qquad (1)$$

where I_1 and I_{SD} are the amplitudes and T_{SD} is the spectral diffusion time constant.

Phase memory time, T_M

The spin-echo decay measurements were carried out by gradually increasing the inter-pulse delay τ of a primary Hahn echo sequence π/2-τ-π-τ-*echo*. With microwave pulses of length $\pi = 32$ ns, strong proton-electron spin modulation was observed. In order to suppress such modulation, microwave pulses of length $\pi = 128$ ns were used. The phase memory time T_M could be deducted by fitting the experimental data to equation (2).

$$I(2\tau)=I(0)\exp[(-2\tau/T_M)^s] \qquad (2)$$

where *s* is a stretching parameter.

DEER measurements

Double Electron-Electron Resonance (DEER) was measured for compound **7.** All DEER experiments used a 4-pulse sequence with ν_1 detection pulses ($\pi/2$ and π) of 32 ns and ν_2 pump pulses of length 16 ns. Data were processed using DeerAnalysis.[7] Using this package, the raw data were smoothed, filtered to remove the effects of electron spin echo envelope modulation (ESEEM) arising from protons, and background-corrected to account for inter-dimer interactions in the three-dimensional homogenous distribution. The sample concentrations were sufficiently small that the background correction was at all times small.

Nano-electrospray ionization mass spectrometry

A sample of 7-rotaxane **10** was diluted to a concentration ~0.5 µM in 90:10 dichloromethane:acetone mixture. A low intensity signal of the intact molecule was observed as a potassium adduct, therefore extra potassium ions were introduced to sample in order to increase its abundance, with the introduction of an aqueous solution of potassium chloride. The concentration of potassium chloride in the sample was adjusted to 10 µM with water content of 0.005% by volume. Measurements were carried out using a Waters Synapt G2 mass spectrometer equipped with a nano-ESI source. Nano-ESI tips were manufactured in-house from borosilicate glass capillaries (Kwik-Fil, World Precision Instruments Inc., Sarasota, FL USA) using a Flaming/Brown Micropipette puller (Model P-97, Sutter Instrumetns Co., Novato, USA). Platinum wire (0.125 mm, 99.95% purity, GoodFellow, Cambridge Ltd, Huntingdon, UK) was used to apply voltage to the sample solution. The capillary voltage was adjusted between 1 and 1.5 kV to optimize spray quality and maximize the abundance of an intact 7-rotaxane ion. The source temperature was set to 60 °C. Sampling cone and extractor cone were set to 35V and 3V respectively. Source gas, and nanoflow pressures were off. Trap gas flow was set to 1.2 mL/min. All the experiments were performed in ToF-MS arrangement in resolution mode. Argon was used as the collision gas for collision induced dissociation experiments. Typically, the scan time was set to 10 seconds and spectra were acquired for 10 minutes. The TriWave settings were as follows:

Trap DC Entrance	0.0
Trap DC Bias	2.0
Trap DC	0.0
Trap DC Exit	2.0
IMS DC Entrance	-20.0
Helium Cell DC	1.0
Helium Exit	-20.0
IMSBias	2.0
IMS DC Exit	20.0
Transfer DC Entrance	5.0
Transfer DC Exit	15.0
Source Wave Velocity (m/s)	300
Source Wave Height (V)	0.0
Trap Wave Velocity (m/s)	300
Trap Wave Height (V)	0.5
IMS Wave Velocity (m/s)	1000
IMS Wave Height (V)	15.4

The mass spectrum revealed species at 5188 m/z (+3) and 3900 m/z (+4) which could correspond to an intact 7-rotaxane **10** ion with 2 and 3 potassium adducts respectively (Supplementary Figure 6). In order confirm the species are the fully assembled 7-rotaxane **10**, a mass selected collision induced experiment was performed. The 5188 m/z ion was selected in the quadrupole mass filter (resolution settings LM=14, HM=14) and collided with argon gas molecules in the ion trap. Fragmentation spectra of 5188 m/z ion are presented in Supplementary Figure 7.

Fragments of the 5188 m/z ion appear in three distinctive regions of the mass spectrum. Proposed peak assignments are detailed in the Supplementary Figures 8-10.

A mass selected CID experiment reveals that the triply charged ion at 5188 m/z fragments into doubly charged species at 5312 m/z and singly charged species at 4937 m/z. These species were assigned in Supplementary Figure 10, as a {Fe_2Co} triangle core with two and one “arms” respectively. Fragment peaks in the range 2000-3000 m/z were assigned as complexes of the thread **C** and {Cr_7Zn} ring(s), presenting with a variety of adducts/losses of potassium, zinc and pivalate (Supplementary Figure 9). A singly charged {Fe_2Co} triangle core was observed at 782 m/z. Its fragments as well as the fragments of the thread **C** were observed in the range 300-500 m/z (Supplementary Figure 10). {Fe_2Co} triangle core fragments were distinguished from those originating from the thread by comparing the isotopic distribution signatures, which are readily different for ions containing transition metals. Therefore, by mass selecting 5188 m/z ion fragmenting it at increasing collision energies, we observe all the building blocks of the 7-rotaxane **10** and their complexes with the correct stoichiometries. Thus we conclude that the peak at 5188 m/z is a fully assembled 7-rotaxane **10** with two potassium adducts. The presence of the same species in the +4 charge state, supports this conclusion further.

Small Angle X-ray Scattering (SAXS)

SAXS measurements were performed on a HECUS SAXS/GISAXS instrument equipped with XENOCS micro focus Cu K-α (λ = 1.5407Å) source equipped with Montel optics and the diffracted X-rays collected with a Dectris Pilatus 100K 2D detector. Samples were dissolved in toluene and contained in borosilicate capillaries with diameter of 1.5mm and wall thickness of 10 μm. Silver behenate was used for calibration of the instrument before every collection. Pure solvent collections were performed with identical conditions as the samples to allow consistent subtraction. Sample collections typically took 10000 seconds. All experimental data are the sum of the 2D radial distribution of the small angle X-ray diffraction converted to a 1D line graph. Irena SAS/SANS routines[8] in Wavemetrics Igor Pro have been used for calibration, data conversion and subsequent analysis.

The analysis involved subtracting the solvent contribution from the sample + solvent data and then employing routines in Irena for the analysis. Pair distance distribution functions provided a reliable, simple and reproducible means for investigating the molecular sizes. The corrected data was analysed using the Moores method (S6). Initially the approximate size is determined and then function fitted to a region between large aggregate signals (small angles) and the statistically insignificant data at high angles. Fitting was repeated until a steady maximum size was achieved.
Comparison to a predicted PDDF calculated from a crytallographically determined structure, was achieved by employing CRSYSOL[S9] from the ATSAS library to

convert a structure PDB for the [4] rotaxane, into a calculated small angle diffraction curve. This was then used to calculate a PDDF curve employing the same routines as for the experimental data. The PDDF's were also compared to those obtained by using the software GNOM[S10] from the ATSAS library and were found to be comparable.

SAXS Analysis and instrumentation Comparison

Below we demonstrate the process that we have employed to assess the viability of employing SAXS as an indicative tool in organo-metallic synthesis.

Instrument Capability

20 nm gold nanoparticles as a standard (see Supplementary Figure 11)

The raw data for gold nano particles provides a clear test of both the instrument capability and analysis employing Irena routines. Although, there is a possibility that aggregation may be occurring at low q the Guinier plot is a good fit to a straight line. This region can also be fitted to a Porod plot with q^{-4} which would be consistent with a smooth surface. However, subsequent collections from clean solvents often display a similar gradient at low q and this contribution has not been subtracted from this data and solvent influence cannot be ruled out. The PDDF data indicates a maximum extent that is consistent with the expected size of the particles. There is a discrepancy for the R_g between the guinier plot (75Å) and values determined from the PDDF (67Å), which seems to consistently provide a smaller value in subsequent analysis. This could also be indicative of some form of aggregation[11] with the PDDF results producing a more reliable R_g.

Comparison of Analysis employing Irena routines (see Supplementary Figure 13)

The analysis employing Irena shows some differences compared to that from Primus and GNOM. There is a possibility that the Guinier plot may show some aggregation. This may explain the discrepancy between in the R_g calculated from the Guinier equation in the two examples of 10.5 Å to 11.5 Å. However, the R_g determined when determining the PDDF in Irena provides the same R_g. Importantly, the PDDF's from both sets of analysis are very similar and yield comparable maximum extent which approximately match the size of the molecule which has been determined using single crystal diffraction.

Comparison of Synchrotron to Laboratory Collection (Supplementary Figure 14)

The simulated data are in quite good agreement with the experimental data. The pair-distribution function (PDDF) from raw scattering data (Supplementary Figure 14b) features 5 Å, 12 Å, 19 Å and 29 Å peaks showing some consistency with the model predictions. The R_g obtained using the Guinier equation (Supplementary Figure 14d, $R_{g\ exp}$ = 15.1 Å) is larger than that from the simulated PDF ($R_{g\ model}$ = 14.3 Å) (Supplementary Figure 14c). The latter value is slightly smaller than that obtained from the experimental PDF ($R_{g\ exp,\ \mathrm{GNOM}}$ = 16.0 Å).

The results are comparable to those obtained using primus and GNOM. The R_g obtained from the Guinier plot (14.699Å) is slightly larger than that determined from the PDDF analysis (13.789Å). These values obtained using Irena compare well to those using Primus/GNOM of 15.1 Å and 14.3 Å.

Comparison of data collected from synchrotron to laboratory source is very favourable although signal to noise is a poorer and q range reduced from the laboratory source as expected.

Two more examples of laboratory data collections from large molecules are shown in Supplementary Figure 16. In the first example a crystal structure is available for a Pd_{12} cage and the overall size can be confirmed. In the second example a proposed molecule is shown from a synthesis and SAXS has provided evidence that the synthesis has been successful. The expected size data is complemented by high resolution high mass spectrometry and also atomic force microscopy which has imaged structures of a consistent size.[12]

Potential Structural Analysis

The nature of the molecules studied is conducive to rigid body modelling and may provide further information when coupled with improved data that needs to be collected from a synchrotron. From the data that we have already collected there is typically some features that are present in the raw data around 0.3-0.4 Å which yields a peak in PDDF at around 10-12 Å. This is demonstrated in Supplementary Figure 17, which displays data for a molecule containing only a single {Cr_7Ni} wheel. Early attempts at employing the modelling routines in Irena have shown that a flattened sphere best matches the data for a single wheel and when coupled with other rigid bodies it is possible to simulate the data.

Supplementary References

1. Nowell H., Barnett S.A., Christensen K.E. and Teat S.J., Allan D.R. I19, the small-molecule single-crystal diffraction beamline at Diamond Light Source. *J. Synchrotron Radiat.*, **19**, 435– 441 (2012).
2. Sheldrick, G.M. SADABS Empirical absorption correction program based upon the method of Blessing. University of Göttingen, 1995,
3. Blessing, R.H. An empirical correction for absorption anisotropy. *Acta Crystallogr.*, **A51**, 33–38 (1995).
4. Sheldrick, G.M. A short history of SHELX. *Acta Crystallogr.*, **A64**, 112–122 (2008).
5. PLATON, A Multipurpose Crystallographic Tool, Utrecht University, Utrecht, The Netherlands (2008).
6. Piligkos, S. *et al.* EPR Spectroscopy of a Family of $Cr^{III}_7M^{II}$ (M = Cd, Zn, Mn, Ni) “Wheels”: Studies of Isostructural Compounds with Different Spin Ground States. *Chem. Eur. J.*, **15**, 3152–3167 (2009).
7. Jeschke, G. *et al.* DeerAnalysis2006–a comprehensive software package for analyzing pulsed ELDOR data. *Appl. Magn. Reson.* **30**, 473 (2006).
8. “Irena: tool suite for modeling and analysis of small-angle scattering", Ilavsky, J. and Jemian, P. R. *J. Appl. Cryst.* **42**, 347-353 (2009).
9. Svergun D.I., Barberato C. and Koch M.H.J. CRYSOL - a Program to Evaluate X-ray Solution Scattering of Biological Macromolecules from Atomic Coordinates *J. Appl. Cryst.* , **28**, 768-773 (1995).
10. Svergun D.I. Determination of the regularization parameter in indirect-transform methods using perceptual criteria. *J. Appl. Crystallogr.* **25**, 495-503 (1992).
11. Putnam, C. D., Hammel, M., Hura, G. L. and Tainer, J. A. X-ray solution scattering (SAXS) combined with crystallography and computation: defining

accurate macromolecular structures, conformations and assemblies in solution. *Quart. Rev. Biophys.* **40**, 191-285 (2007).

12. Ferrando-Soria J. *et al.* Controlled synthesis of nanoscopic metal cages. *J. Am. Chem. Soc.* **137**, 7644-7647 (2015).
13. Harris, K., Sun, Q.-F., Sato, S. & Fujita, M. $M_{12}L_{24}$ Spheres with Endo and Exo Coordination Sites: Scaffolds for Non-Covalent Functionalization. *J. Am. Chem. Soc.* **135**, 12497-12499 (2013).
